# Loss of Brain‐Derived Estrogen Is Associated With Sex‐ and Age‐Dependent Alterations in Memory, Affective Behavior, and Hippocampal Extracellular Matrix Gene Expression

**DOI:** 10.1111/acel.70551

**Published:** 2026-05-26

**Authors:** Natalie C. Piehl, Ariel W. Halle, Guadalupe Rodriguez, Andrea Locci, Stacy Kujawa, Caroline Haywood, John Coon, Ross P. McNally, Zaina A. Karim, Tianming You, Hongxin Dong, Serdar E. Bulun, Hong Zhao

**Affiliations:** ^1^ Division of Reproductive Science in Medicine, Department of Obstetrics and Gynecology, Feinberg School of Medicine Northwestern University Chicago Illinois USA; ^2^ Department of Psychiatry & Behavioral Sciences, Feinberg School of Medicine Northwestern University Chicago Illinois USA

**Keywords:** aging, Alzheimer's disease, aromatase, estrogen, extracellular matrix, memory

## Abstract

Nearly two‐thirds of Americans with Alzheimer's disease (AD) are women. Prior research suggested that women with AD have lower brain estrogen levels than those without AD. However, how estrogen deficiency modulates this sex‐based difference in AD vulnerability is not well understood. Aromatase, the key enzyme for estrogen biosynthesis, is expressed in both neurons and astrocytes of the brain, including the hippocampus. This study aims to assess the mechanistic link between brain‐selective aromatase deficiency and sex‐specific AD vulnerability. To achieve this goal, we used brain‐specific aromatase knockout (*bArKO*) and whole‐body total aromatase knockout (*tArKO*) mice of both sexes at young (6‐ to 8‐month‐old) and old (> 19‐month‐old) ages. We found that aromatase deletion decreased brain estrogen levels in *bArKO* mice and circulating and brain estrogen levels in *tArKO* mice. Impairment in spatial working memory and social interaction behavior was observed only in old female *bArKO* and *tArKO* mice. Both young and old female, but not male, *tArKO* mice displayed depression‐like behavior. Bulk RNA‐seq analysis of hippocampal tissues from young and old *bArKO* mice of both sexes revealed enrichment of extracellular matrix‐related pathways and upregulated mRNA and/or protein expression of extracellular matrix‐associated genes (e.g., *Col1a1*, *Ccn2*, *Dcn*, and *Ogn*) in old female *bArKO* mice compared to littermate control mice. These findings point to a novel link between local brain estrogen deficiency and sex‐ and age‐specific extracellular matrix changes in the hippocampus of old *bArKO* female mice accompanied by AD‐related memory and behavioral impairments.

## Graphical abstract

Age, sex, and estrogen loss are widely recognized risk factors for Alzheimer's disease. Here, we show that brain‐specific aromatase knockout (*bArKO*) and the resulting estrogen loss lead to memory impairment, reduced social interaction, and extracellular environment dysregulation selectively in aged female mice, but not in young female or male mice.

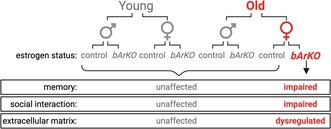

## 1 Introduction

Currently, over 7 million people 65 years of age and older in the US are living with Alzheimer's disease (AD), a number that is projected to reach 14 million by 2060 (Rajan et al. 2021). Nearly two‐thirds of Americans with AD are women (Alzheimer's Association 2025; Gaugler et al. 2019); however, the underlying molecular mechanisms for this sex difference in AD vulnerability are largely unknown. A body of evidence suggests that strikingly decreased levels of circulating estradiol (E2, the most potent estrogen) after menopause lead to a loss of its neuroprotective functions in the brain (Fisher et al. 2018; Paganini‐Hill and Henderson 1994), although the results of clinical studies of hormone replacement therapy (HRT) have been contradictory. A large number of studies have reported either no effects (Henderson et al. 2000; Mulnard et al. 2000) or benefits of E2 replacement on the prevention of AD (Asthana et al. 2001; Paganini‐Hill and Henderson 1994; Tang et al. 1996). The most frequently quoted results come from the Women's Health Initiative Memory Study, which reported an approximately 2‐fold increased risk of dementia/AD if HRT was initiated in predominantly comorbid postmenopausal women after 65 years of age (Rapp et al. 2003; Shumaker et al. 2003). These findings have been attributed to destabilization of atherosclerotic plaques by late estrogen exposure, leading to neurodegeneration of vascular origin. Other studies have shown that early estrogen exposure immediately after menopause is beneficial by exerting anti‐inflammatory effects on brain tissues and vasculature (Arnal et al. 2009; Greendale et al. 2011). Further studies suggest HRT may be beneficial for cognition and mood disorders when given to symptomatic women within 10 years of the onset of menopause or under 60 years of age (Girard et al. 2017; Speth et al. 2018). HRT has been estimated to reduce the risk of dementia or AD by approximately 11%–33%, depending on HRT type, age at initiation, and study design (Stute et al. 2021). However, it remains unclear why the timing of HRT initiation is critical to its effects on the brain, and what mechanisms underlie the protective effect of HRT against the risk of AD in women. Mechanistic studies focused on the sex‐specific impact of estrogen on brain function are essential for developing better HRT strategies that can help prevent or slow the progression of AD in women.

Aromatase, the essential enzyme for E2 biosynthesis, converts androgen into estrogen (Bulun et al. 2005; Zhao et al. 2016). In premenopausal females, the ovary serves as the primary source of circulating (systemic) estrogen. Following menopause, ovarian estrogen production declines sharply, and extremely low levels of circulating estrogen are maintained largely through local synthesis in multiple other tissues, including the brain, bone, adipose tissue, muscle, breast, and vasculature in humans. In mice, estrogen is locally synthesized in the brain and gonadal fat in males, whereas in females it is produced predominantly in the brain (Bulun et al. 2005; Zhao et al. 2009). Aromatase is expressed in both neurons and astrocytes of the brain, with high levels in the hippocampus (Azcoitia et al. 2003; Luchetti et al. 2011; Yague et al. 2006), a region critical for memory and cognition (Kretz et al. 2004; Sato and Woolley 2016). Hippocampal E2 plays key roles in neuronal protection and synaptogenesis (Kretz et al. 2004; Manaye et al. 2011; Mukai et al. 2010), and hippocampal aromatase expression and brain E2 levels are lower in women with AD compared to those without AD (Ishunina et al. 2007; Li et al. 2014). Local infusion of an aromatase inhibitor into the dorsal hippocampus of female mice impairs consolidation of spatial or object recognition memory (Tuscher et al. 2016). Additionally, β‐amyloid (Aβ) in the hippocampus is significantly exacerbated by whole‐body aromatase knockout (*ArKO*) in AD (APP23) female mice (Yue et al. 2005) and by subcutaneous administration of an aromatase inhibitor in female but not male AD (3xTg) mice (Overk et al. 2012). Conversely, administration of E2 or the selective ERα agonist propylpyrazoletriol (PPT) ameliorates Aβ accumulation and improves working memory in 3xTg‐AD mice (Carroll and Pike 2008; Carroll et al. 2007; Koss and Frick 2019). Although a beneficial effect of estrogen on female brain function and memory has been observed in many contexts, the underlying mechanism by which estrogen signaling influences memory function and sex‐specific vulnerability to AD in women remains poorly understood.

Conventional whole‐body *ArKO* mice exhibit depletion of estrogen production in all tissues, resulting in infertility and dysregulation of systemic E2, testosterone, and gonadotropins (FSH and LH) from a young age, making it an unsuitable model to study the specific contribution of brain estrogen to sex differences in AD (Fisher et al. 1998). Additionally, the reduced estrogen production in reproductively senescent female humans and mice makes young mice an insufficient model to address the effects of estrogen in the brain. Therefore, we used aromatase knockout mice, either selectively in the brain (*bArKO*) or totally in the whole body (*tArKO*) to study the roles of local brain estrogen in brain function and the underlying molecular mechanisms. We compared memory and AD‐related affective behaviors in the *bArKO* and *tArKO* models and their littermate controls, comparing males and females at both young and old ages. We demonstrated that brain aromatase is critical for memory and affective behavior in old female mice but not old male mice. Through bulk RNA‐seq analysis of the hippocampus of *bArKO* mice, we further revealed a potential molecular mechanism underlying sex‐specific memory impairment.

## 2 Materials and Methods

### 2.1 *bArKO* And *tArKO* Mouse Generation and Maintenance

The floxed aromatase gene (*Arom* ^*fl/fl*^) mouse (C57BL/6J background) was generated and genotyped in our laboratory, as previously described (Brooks et al. 2020). Transgenic *Nestin‐Cre* mice (#3771) and *Zp3‐Cre* mice (#3651) on a C57BL/6J background were obtained from The Jackson Laboratory and crossed with *Arom* ^*fl/fl*^ mice to generate *bArKO* or *tArKO* mice, respectively. Mice were maintained on a 14‐h light:10‐h dark cycle with standard chow (7912; Harlan Teklad) and water *ad libitum*. All animal experiments and husbandry were approved by and conducted in accordance with guidelines established by the Institutional Animal Care and Use Committee at Northwestern University. The data from animal experiments were analyzed by investigators blinded to mouse age, sex, and genotype. Age‐matched *Arom* ^*fl/fl*^ mice were used as controls for *bArKO* mice, and wild‐type (WT) mice served as controls for *tArKO* mice.

### 2.2 Serum Hormone Levels Measured by Enzyme‐Linked Immunosorbent Assay (ELISA)

Serum was collected from 8‐ to 26‐week‐old female *bArKO*, *tArKO*, and their littermate control mice via retro‐orbital bleeding. All serum samples were collected from mice between 10 am and noon to minimize assay variability due to daily hormone fluctuations. Serum levels of E2 in *bArKO* and *tArKO* mice were measured by ELISA at the Ligand Assay and Analysis Core of the University of Virginia Center for Research in Reproduction, as described previously (Haisenleder et al. 2011; Zhao et al. 2012).

### 2.3 Behavioral Tests

A series of behavioral tests was conducted for *bArKO*, *tArKO*, and littermate control mice at 6–8 months of age (young) and > 19 months of age (old) with at least 2 days of gap between each test. Mice were handled once a day for five consecutive days before starting the behavioral tests and transferred to the testing room at least 1 h before starting a test (pre‐test acclimation period) to reduce stress levels due to exposure to the new environment. The testing session started at 9 am on every test day. All chambers, tools, and instruments were thoroughly cleaned with 70% alcohol to eliminate olfactory stimuli between any session. The testing sequence was set as follows: (1) open field, (2) novel object recognition, (3) Y‐maze, (4) social interaction and social recognition, (5) light–dark box, and (6) tail suspension test (Locci et al. 2021).

#### 2.3.1 Open Field (OF) Test

The OF test was used to evaluate mouse locomotion and anxiety‐like behavior. The apparatus for the OF test consisted of an evenly illuminated plexiglass box (40 × 40 × 40 cm) placed on a stable table with overhead video recording. Animal activity was recorded using an automated video tracking system (Any‐Maze, Stoelting, Wood Dale, IL). Locomotor activity was determined as the distance traveled (m) during a 10‐min trial (Viana et al. 2013). Anxiety‐like behavior was measured based on the number of times the mouse entered the center (inner third) of the chamber, and the total time spent there. Mice with less anxiety spent more time in the center and mice with more anxiety stayed at the edges of the chamber.

#### 2.3.2 Novel Object Recognition (NOR) Test

NOR tests were used to assess recognition memory. The 3‐day NOR tests were conducted in the same chambers as described for the OF test above. Two sets of objects with consistent sizes (heights and volumes) but different shapes and colors were used. Mice were individually habituated in the empty chamber for 10 min for the first 2 days. On day 3, the experimental session consisted of 3 phases: (1) acquisition trial (10 min); (2) inter‐trial‐interval (home‐cage, 1 h); and (3) retention trial (10 min). During acquisition, mice were videotaped while exploring the chamber containing two identical objects placed diagonally across from each other. After the inter‐trial‐interval, animals were put back in the arena for the retention trial with one of the objects replaced by a novel object with a similar size but a different color and shape in a counterbalanced manner. Exploration of the objects was defined as touching, leaning on the object and sniffing, or orienting the head toward the object and sniffing within < 1.0 cm. Climbing on top of the objects was not counted as exploration. Using two stopwatches, an experimenter blind to the experimental groups recorded the amount of time each mouse spent exploring and the number of interactions with each object during the retention trial. The discrimination index was calculated as the proportion of the difference in the numbers of interactions (entries) and the total time (time) spent exploring between the two objects. A higher discrimination index would indicate better recognition memory.

#### 2.3.3 Y‐Maze Test

The Y‐maze test is used to evaluate spatial working memory on a continuous spontaneous alternation task. The Y‐maze apparatus consists of a three‐arm (Arms A, B, and C, 5 cm wide × 21 cm long × 15.5 cm high) maze with three different special cues positioned in the top inner part of each arm. The apparatus was placed on a stable table with overhead video recording using an automated video tracking system (Any‐Maze). Mice were placed in arm “A” (starting point, excluded by the analysis) facing the end of the arm and then were allowed to freely explore the apparatus for 5 min in the absence of the investigator while a camera recorded their movements. A correct alternation was defined as a sequence of three consecutive entries into different arms of the maze (i.e., ABC, ACB, BAC, BCA, CAB, and CBA) without reentering the two previously visited arms. The percentage of spontaneous alternation was calculated by dividing the number of total successful alternations by the total number of possible alternations (i.e., the number of total entries minus two) multiplied by 100. For selective ERα agonist PPT treatment, female *tArKO* mice at 6–8 months of age were treated with PPT (2.5 mg/kg body weight, intraperitoneal injection) 2 h before the Y‐maze test.

#### 2.3.4 Social Interaction (SI) and Social Recognition (SR) Tests

SI tests are used to assess various aspects of social behavior. SR tests are designed to assess SR memory by distinguishing familiar and novel conspecifics (members of the same species). The SI and SR apparatus chamber (60 cm wide × 60 cm long × 40 cm high) was divided equally into three compartments using removable dividers with a small door that allows the experimental mouse to move between the three compartments. The apparatus was placed on the floor with overhead video recording (Any‐Maze). SI and SR tests were conducted over 3 days (habituation on Day 1, SI on Day 2, and SR on Day 3).

On habituation day, the experimental mouse was freely exposed to the chamber and the three compartments for 10 min. On SI day, an intruder conspecific mouse with the same strain, sex, and age as the experimental mouse was placed in one of the lateral compartments of the test chamber under a cylinder (10 cm circumference, 8 cm high). Experimental mice were naive to their intruder mice before starting the test. In the other lateral compartment, a similar cylinder was placed without a stimulus mouse. The experimental mouse started the session in the central compartment and then was allowed to explore the three compartments freely for 10 min. To quantify SI of the experimental mouse, the amount of time the mouse spent in the chamber with the intruder mouse and the number of interactions with the intruder mouse were scored by an observer blind to the experimental group.

On SR day, the experimental mouse was re‐exposed to the same chamber and two conspecific intruder mice—the same intruder mouse used in the SI test (“old mouse”) and a new intruder mouse (“new mouse”) with the same strain, sex, and age as the old mouse. The experimental mouse was allowed to explore freely and was videotaped for 10 min. The time spent around and the number of interactions with the “old” and “new” mice were used as an index of social recognition.

#### 2.3.5 Light/Dark Box (LD) Test

The LD test is primarily used to assess anxiety‐like behavior. The LD test chamber (60 cm wide ×60 cm long ×40 cm high) was divided equally into two compartments using removable dividers with a small door that allowed the experimental mouse to move from one compartment to another. One compartment was dark (< 5 lx) and the other was illuminated with a bright stimulus light (300 lx). The chamber was placed on a table with overhead video recording. Mice were placed in the light compartment and allowed to explore both dark and light compartments for 10 min. Animal activity was recorded using an automated video tracking system (Any‐Maze). The time spent and the number of entries into each compartment were used to measure the levels of anxiety‐like behavior.

#### 2.3.6 Tail Suspension Test (TST)

The TST test is used to assess depressive‐like behavior in rodents. The detailed TST was performed as previously described (Can et al. 2012). Briefly, mice were suspended for 6 min by applying tape (17‐cm long stripes) onto the distal portion of their tails and subjected to frontal video recording. Videos were scored by a researcher blind to the experimental groups by using a stopwatch. Immobility time was taken as an index of depressive‐like behavior.

### 2.4 RNA Isolation, Library Preparation, and Sequencing for Bulk RNA‐Seq

We performed bulk RNA‐seq on 24 hippocampi collected from 6 young (6 months) and 6 old (24 months) *bArKO* mice plus 12 hippocampi from age‐matched littermate controls of both sexes. Hippocampal RNA was isolated using the RNeasy Mini Kit (Qiagen) and libraries were made using the Illumina TruSeq Stranded mRNA Library Prep Kit (# 20020595). Library quality was assessed using the Agilent 2100 Bioanalyzer. Sequencing was performed on the Illumina HiSeq 4000 Sequencing System at the Next‐Generation Sequencing Core at Northwestern to obtain 25 million single‐end 50‐bp reads per sample.

### 2.5 Analysis of Bulk RNA‐Seq

All tools were used with default parameters unless otherwise specified. Bulk RNA‐seq libraries were assessed for quality using FastQC v0.12.0. Adapters were trimmed from libraries using TrimGalore v0.6.10, a wrapper around Cutadapt v4.2, with stringency set to 5. STAR v2.7.9a was used to align sequences to the GRCm39 genome assembly with the following nondefault parameters: ‐‐outSJfilterCountUniqueMin ‐1 2 2 2, ‐‐outSJfilterCountTotalMin ‐1 2 2 2, ‐‐outFilterIntronMotifs RemoveNoncanonical, ‐‐outFilterMultimapNmax 2, ‐‐outFilterMismatchNmax 10. HTSeq v2.0.2 was used to generate a counts matrix from alignments. All downstream analyses were performed with R v4.1.1. Count matrices were processed using DESeq2 v1.34.0 with design set to sex + genotype (i.e., female_*bArKO*). DESeq was run using the Wald test and a parametric fit and differentially expressed genes (DEGs) were defined using a log‐fold change threshold of 0.50 and a Benjamini–Hochberg corrected *p* value threshold of 0.05. Gene set enrichment analysis (GSEA) and over‐representation analysis were performed using clusterProfiler v4.13 and gene ontology molecular function (GO MF) and cellular component (GO CC) databases.

### 2.6 RNA Isolation and Quantitative Real‐Time PCR (qPCR)

Total RNA was extracted from the hippocampus of old female *bArKO* and control mice (19–23 months, *n* = 3 per group) using RNeasy RNA purification kit (Qiagen). cDNA was reverse transcribed using oligo(dT) primers and a reverse transcription kit (Quanta Biosciences). Real‐time qPCR was performed using the Taqman or the Power SYBR green PCR master mix kit (Applied Biosystems) on an ABI QuantStudio 5 K Real‐Time PCR System (Applied Biosystems). The SYBR green‐based QuantiTect mouse primers were from Qiagen (*Esr1* [ERα], *Esr2* [ERβ], and *Gper1*). The Taqman‐based primers were purchased from Thermo Fisher Scientific (*Col1a1*) or IDT (*Col1a2, Vim, Ccn2, Dcn, Ogn*, and *Gapdh*). Real‐time PCR cycler conditions were 50°C for 2 min, 95°C for 10 min, and 40 cycles of 95°C for 15 s and 60°C for 1 min. qPCR results were normalized to mouse *Gapdh* gene expression. Ct values at or above 40 cycles were considered to be below the level of detection.

### 2.7 Protein Extraction and Immunoblotting

Total protein from the hippocampal tissue of 3 male controls, 3 male *bArKO* mice, 3–4 female controls, and 3–4 female *bArKO* mice, 4 female WT mice, 4 female *tArKO* mice (~20 months) was extracted in RIPA buffer containing protease inhibitors (2 mmol/L phenylmethylsulfonyl fluoride, 10 mg/mL leupeptin, and 10 mg/mL aprotinin) and phosphatase inhibitors (100 mmol/L sodium fluoride, 10 mmol/L sodium pyrophosphate, and 2 mmol/L sodium orthovanadate). Tissue and buffer were ground for approximately 10 s on ice using a VWR Pellet Mixer (#47747–370) and then incubated on ice for 30 min. Lysates were centrifuged at 21,000 x g for 15 min at 4°C. Protein concentrations were determined using the BCA protein assay kit (Pierce). 30 μg of protein were heated at 80°C for 10 min in LDS Sample Buffer (Thermo Fisher Scientific) containing 5% β‐mercaptoethanol and subjected to electrophoresis on 4%–12% NuPAGE Bis‐Tris‐polyacrylamide precast gels (Thermo Fisher Scientific). Proteins were then transferred from gels to PVDF membranes. The membranes were blocked with 5% nonfat milk in TBS‐Tween 20, and proteins were detected using various antibodies (ERα [04‐820, EMD Millipore, 1:2000]; CCN2 [PA5‐32193, Thermo Fisher Scientific, 1:1000]; DCN [14667‐1‐AP, Proteintech, 1:1000]; OGN [12755–1‐AP, Proteintech, 1:1000]; SLC17A6 [135,403, Synaptic Systems, 1:1000]; TGFBR2 [PA5‐35076, Thermo Fisher Scientific, 1:1000]; WNT1 [27935‐1‐AP, Proteintech, 1:500]). After extensive washing, immune complexes were detected with horseradish peroxidase conjugated with specific secondary antibodies (antirabbit [Cell Signaling Technology] or antisheep [Invitrogen]), followed by enhanced chemiluminescence reactions. Blots were analyzed by densitometry and quantified with the ImageJ software.

### 2.8 Transcardial Perfusion and Immunofluorescence Staining

Deeply anesthetized aged *bArKO*, *tArKO*, and WT control mice by intraperitoneal injection of ketamine/xylazine (100/10 mg/kg) were transcardially perfused with 1× PBS until outflow ran clear, followed by 4% paraformaldehyde for 5 min after hindlimb stiffening was observed. Brain tissues were collected and fixed in 4% paraformaldehyde at 4°C for 48 h, followed by cryoprotection in 30% sucrose solution until fully dehydrated (~24 h). Tissues were embedded in Tissue‐Tek OCT compound (Electron Microscopy Sciences) and sectioned at 20 μm thickness using a cryostat (Leica CM 1850 UV). Sections with hippocampal CA1, CA3, and DG regions were selected for immunofluorescence staining. Sections were permeabilized with 0.5% Triton X‐100 in PBS for 30 min and then blocked with 1% BSA and 0.5% Triton X‐100 in PBS for 1 h at room temperature to reduce nonspecific binding. Primary antibodies were diluted in blocking solution and incubated with sections overnight at 4°C (CCN2 [ab6992, Abcam, 1:500]; DCN [14667‐1‐AP, Proteintech, 1:200]; SLC17A6 [135,403, Synaptic Systems, 1:500], GFAP [ab4674, Abcam, 1:1000], NeuN [266,004, Synaptic Systems, 1:1000]). After washing with PBS, sections were incubated with appropriate fluorophore‐conjugated secondary antibodies for 1 h at room temperature in the dark. Following final washes, sections were mounted using antifade mounting medium containing DAPI for nuclear staining. Images were acquired using a Nikon SoRA system (NIH 1S10OD032270‐01) at the Center for Advanced Microscopy (RRID:SCR_020996) funded by the Robert H Lurie Comprehensive Cancer Center (CCSG P30 CA060553). SLC17A6, CCN2, and DCN immunofluorescence signals were quantified in the CA1 region by average intensity in ImageJ (NIH). Colocalization was measured using Mander's Coefficient after thresholding each channel using mean + 3.5*SD. Images were processed and quantification was performed blindly.

### 2.9 Statistical Analysis

Results are expressed as mean ± s.e.m., unless otherwise indicated. Statistically significant differences at *p* < 0.05 were determined using two‐tailed Student's *t*‐test, Mann–Whitney test, one‐way ANOVA, two‐way ANOVA, or Kruskal–Wallis test. Except for bulk RNA‐seq analysis, all other statistical tests were performed using the GraphPad Prism software.

## 3 Results

### 3.1 Aromatase Expression Is Abolished in the Brain of *bArKO* Mice and in the Whole Body of *tArKO* Mice

**FIGURE 1:**
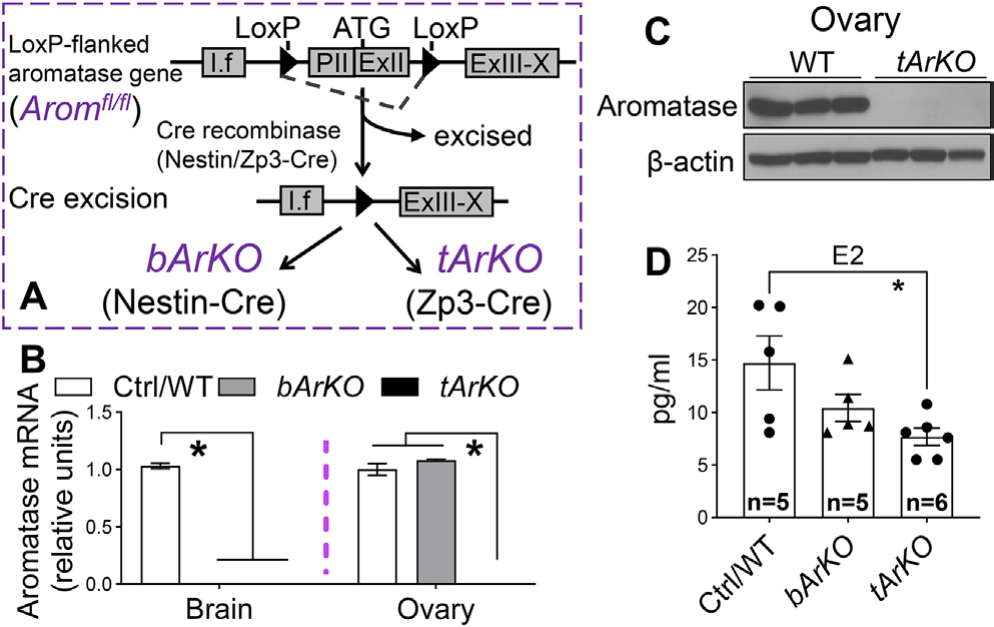
Generation and characterization of female *bArKO* and *tArKO* mice. (A) Schematic demonstrating generation of *bArKO* and *tArKO* mice. *Nestin‐Cre* and *Zp3‐Cre* mice were used to generate *bArKO* and *tArKO* mice, respectively. I.f, brain‐specific exon 1. PII, gonad‐specific first exon. ExII, exon 2. ExIII‐X, exons 3–10. (B) qPCR analysis of aromatase‐expressing tissues in female control (Ctrl)/wild‐type (WT), *bArKO*, and *tArKO* mice. *Gapdh* mRNA levels served as loading controls. *n* = 5. One‐way ANOVA with Tukey's multiple comparison test, *p < 0.05. (C) Immunoblotting showed deletion of aromatase protein in the ovary of *tArKO* female mice. β‐Actin served as a loading control. *n* = 3. (D) Serum E2 levels were measured in female *bArKO* and *tArKO* mice. Serum levels from 12‐week‐old mice were measured by ELISA. The number of mice in each group is indicated in each column. One‐way ANOVA with Tukey's multiple comparison test, *p < 0.05.

*bArKO* and *tArKO* male mice were generated and characterized as previously described (Brooks et al. 2020). Briefly, we first generated mice with a loxP‐flanked aromatase gene (*Arom* ^*fl/fl*^) to knockout aromatase in a tissue‐specific fashion. This floxed region includes the transcription and translation start sites and the common splice acceptor site for the upstream brain aromatase promoter I.f (Figure 1A). We crossed *Nestin‐Cre* mice with *Arom* ^*fl/fl*^ mice to generate brain‐specific aromatase knockout mice (*bArKO*) (Brooks et al. 2020). We also generated whole‐body total aromatase knockout mice (*tArKO*) by crossing the same *Arom* ^*fl/fl*^ mice with *Zp3‐Cre* mice (Brooks et al. 2020). To characterize *bArKO* and *tArKO* female mice, aromatase mRNA in the mouse brain and ovary was analyzed by real‐time qPCR (Figure 1B). In *bArKO* female mice, aromatase mRNA was reduced by more than 100‐fold in the brain compared to control (*Arom* ^*fl/fl*^) female mice, whereas no difference was detected in the ovary. As expected, aromatase mRNA was undetectable in both the brain and ovary of female *tArKO* mice. Further, aromatase protein was also undetectable in the ovaries of *tArKO* female mice (Figure 1C). These findings in female *bArKO* and *tArKO* mice mirror previously published findings in male *bArKO* and *tArKO* mice (Brooks et al. 2020). Briefly, aromatase mRNA was lower in the brain but not in other aromatase‐expressing tissues (testis, epididymis, or gonadal fat) of male *bArKO* mice compared to control mice, and was undetectable in all tissues collected from male *tArKO* mice.

We further characterized the female *bArKO* and *tArKO* mice for reproductive function and fertility, compared to our previously published findings in male *bArKO* and *tArKO* mice (Brooks et al. 2020). Similar average litter sizes were noted in controls and *bArKO* male mice (Brooks et al. 2020); here, we found that female *bArKO* mice also had the same average litter size compared to control females (Figure S1A). Also similar to that previously reported in male *tArKO* mice (Brooks et al. 2020), we observed infertility in all female *tArKO* mice (Figure S1B). Body weight was assessed weekly for female *bArKO*, *tArKO*, and age‐matched littermate control mice from 3 to 48 weeks of age. The rate of increase in body weight from week 3 to 48 was similar between female control and *bArKO* mice (Figure S1C), similar to that reported for *bArKO* males (Brooks et al. 2020). In female *tArKO* mice, however, the rate of body weight increase began to significantly accelerate compared to WT mice starting at 12 weeks of age (Figure S1D); this acceleration was reported to begin at 20 weeks in *tArKO* males (Brooks et al. 2020). Together, these phenotypes indicate that the aromatase gene was functionally deleted in the brain of *bArKO* mice and the whole body of *tArKO* mice.

### 3.2 Aromatase Deletion Decreases Brain E2 Levels in *bArKO* Mice and Circulating, Brain, and Testicular E2 Levels in *tArKO* Mice

To determine whether deletion of brain aromatase alters systemic and tissue estrogen levels in adult mice, we measured serum and tissue E2 levels using liquid chromatography–tandem mass spectrometry (Brooks et al. 2020) or ELISA in *bArKO* mice. Serum E2 levels were similar in female (Figure 1D) *bArKO* mice and their littermate controls, similar to that reported for *bArKO* male mice (Brooks et al. 2020). We previously also showed that brain, but not testis, E2 levels were significantly lower in male *bArKO* mice compared to control males (Brooks et al. 2020). Additionally, *tArKO* mice showed a more severe decrease in serum and tissue E2 levels than *bArKO* mice, with lower serum E2 levels in both female (Figure 1D) and male *tArKO* mice (Brooks et al. 2020) and lower brain and testis E2 levels in male *tArKO* (Brooks et al. 2020) mice compared to their WT male littermates. These results suggest that brain‐specific aromatase deletion diminishes locally produced E2 in the brain and whole‐body aromatase deletion diminishes both serum and tissue estrogen.

### 3.3 Impairment of Spatial Working Memory in Old Female *bArKO* and *tArKO* Mice

**FIGURE 2:**
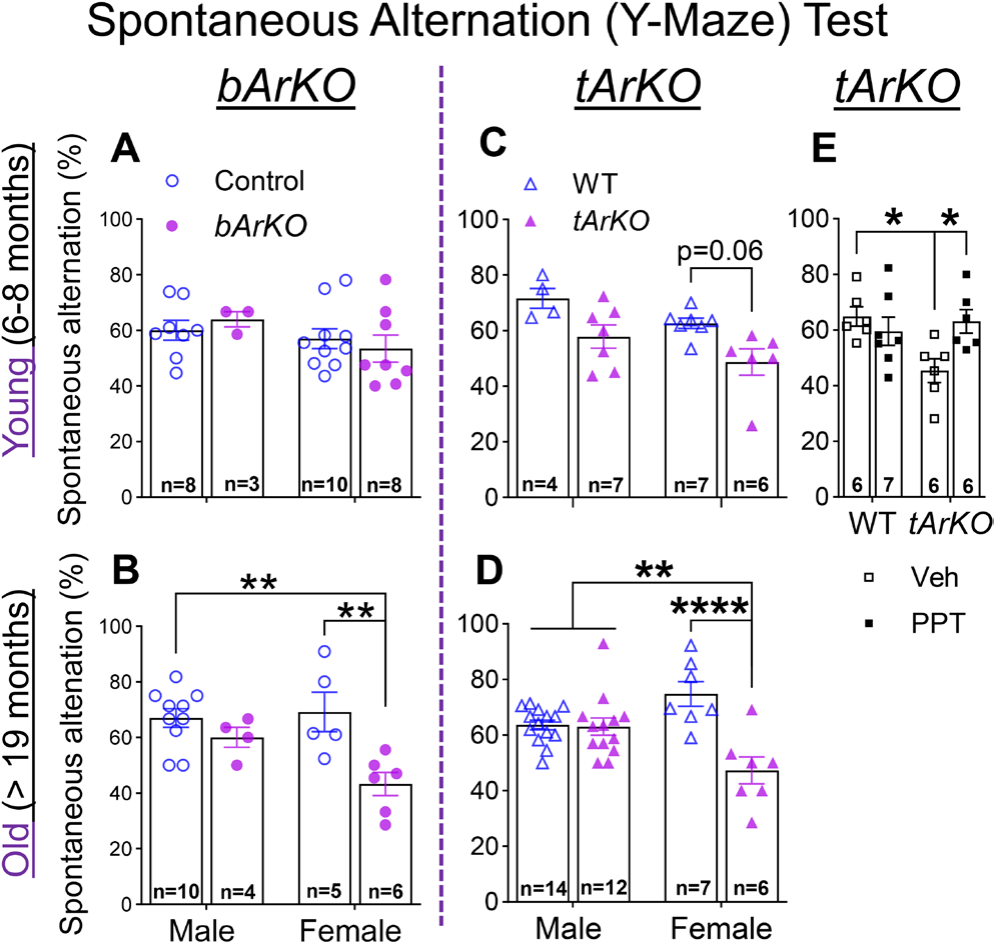
Spontaneous alternation (Y‐maze) test shows impaired spatial working memory in old female *bArKO* and *tArKO* mice. Y‐maze tests were performed in young and old *bArKO* and *tArKO* mice of both sexes. The percentage of spontaneous alternations indicating spatial working memory was calculated for young (A) and old (B) *bArKO* mice and young (C) and old (D) *tArKO* mice. **p < 0.01, ****p < 0.0001. (E) Treatment of selective ERα agonist PPT improves working memory in young female *tArKO* mice. PPT (2.5 mg/kg body weight, intraperitoneal injection) was given 2 h before the Y‐maze test. *p < 0.05. The number of mice used in the test is indicated in each column. Two‐way ANOVA with Tukey's multiple comparison test was used.

A spontaneous alternation (Y‐maze) test was used to determine whether an interaction among estrogen deficiency, age, and/or sex affects spatial working memory in young and old *bArKO* and *tArKO* mice and their control littermates of both sexes. We found that the percentage of spontaneous alternations was lower in old but not young female *bArKO* mice compared to age‐matched control littermates (Figure 2A,B). Correlating with the severe deficiencies in both serum and tissue E2, young *tArKO* female mice exhibited a trend toward reduced spatial working memory (*p* = 0.06), which became significantly impaired in old female *tArKO* mice compared with female WT littermates (Figure 2C,D). Working memory was not impaired in male *bArKO* or *tArKO* mice at any age. Furthermore, we treated young adult *tArKO* female mice at 6–8 months of age with a selective ERα agonist PPT (2.5 mg/kg body weight, intraperitoneal injection) 2 h before the Y‐maze test (Phan et al. 2011). The percentage of spontaneous alternations was decreased in vehicle‐treated *tArKO* female mice but returned to normal levels (as in WT) after PPT treatment (Figure 2E). These results indicate that ERα action in the brain is related to improving memory in females. Novel object recognition memory indicated by discrimination index was not different in old *bArKO* and *tArKO* mice of both sexes compared to sex‐matched littermate controls (Figure S2). Social recognition memory suggested by the number of interactions with or the time spent around the old and new conspecific also was not different in old *bArKO* and *tArKO* mice of both sexes compared to control and WT mice, respectively (Figure S3). Thus, estrogen deficiency in the brain affects spatial working memory in a sex‐specific manner, with impairment in old female but not old male *bArKO* mice. Novel object recognition and social recognition memory may not be affected by brain estrogen deficiency.

### 3.4 Young and Old Female *tArKO* Mice Display Depression‐Like Behavior

**FIGURE 3:**
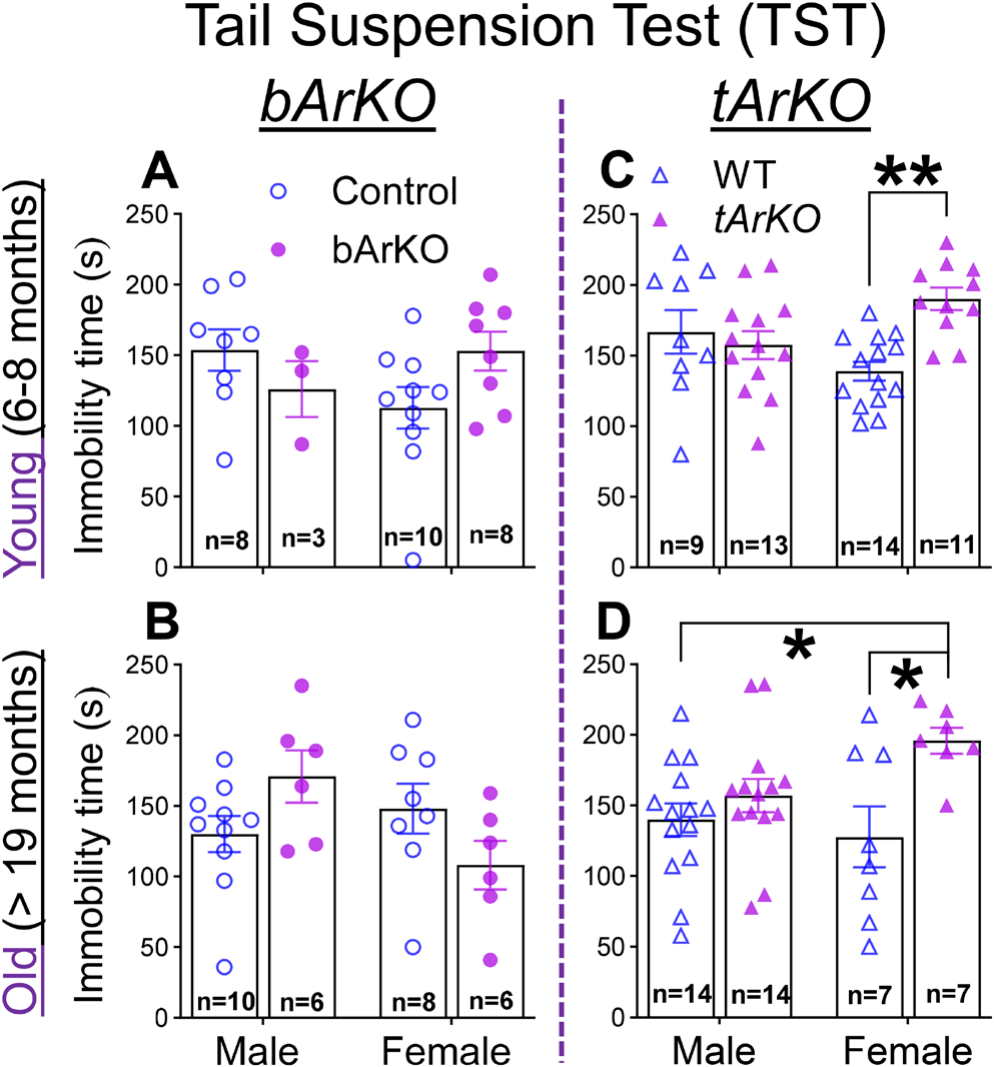
Female *tArKO* mice exhibit depression‐like behavior independent of age. Tail suspension tests (TST) were performed in young (A) and old (B) *bArKO* mice and young (C) and old (D) *tArKO* mice of both sexes. Prolonged immobility suggests depression‐like behavior. The number of mice used in TST testing is indicated in each column. Two‐way ANOVA with Tukey's multiple comparison test was used, *p < 0.05, **p < 0.01.

Since dysregulation of affective behavior (e.g., depressive‐like, anxiety‐like, and social behavior) is associated with memory impairment in AD, we first evaluated depression‐like behavior in *bArKO* and *tArKO* mice using the tail suspension test (TST) (Can et al. 2012). Young and old *bArKO* mice did not show a significantly higher immobility time compared to age‐matched control mice of both sexes (Figure 3A,B). However, young and old *tArKO* female mice showed higher immobility time compared to age‐matched WT female mice, suggesting greater depression‐like behavior (Figure 3C,D). *tArKO* male mice did not show depression‐like behavior. Thus, a decrease in systemic estrogen levels in *tArKO* mice was associated with depression‐like behavior in a sex‐specific manner in both young and old female mice.

### 3.5 Old Female *bArKO* and *tArKO* Mice Display Impaired Social Behavior

**FIGURE 4:**
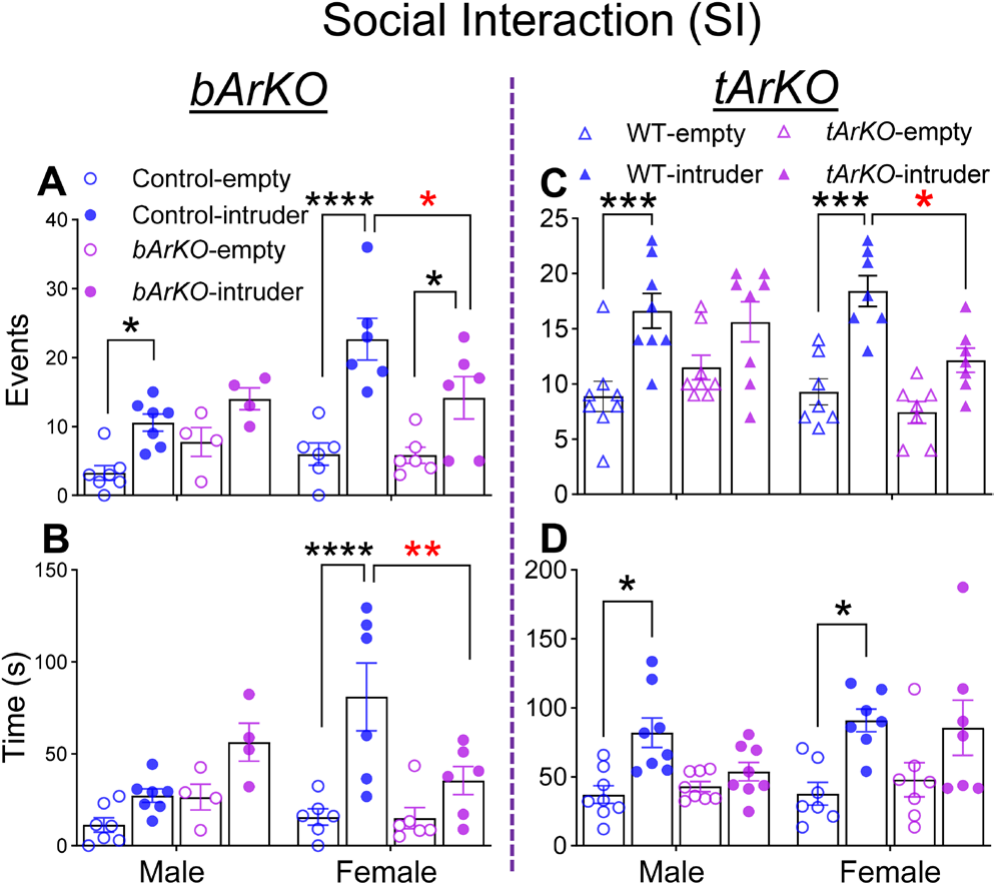
Brain‐specific or total aromatase knockout impairs social behavior in old female mice. Social interaction (SI) tests were performed with old *bArKO* (A and B) and old *tArKO* (C and D) mice of both sexes at age > 19 months. Old *bArKO* and *tArKO* female mice interacted fewer times with and/or spent less time with conspecific intruder mice, suggesting impaired social behavior. For *bArKO* mice, *n* = 7 for control males, *n* = 4 for *bArKO* males, and *n* = 6 for control and *bArKO* females. For *tArKO* mice, *n* = 8 for male and *n* = 7 for female *tArKO* and WT control mice. Two‐way ANOVA with Tukey's multiple comparison test was used, *p < 0.05, **p < 0.01, ***p < 0.001, ****p < 0.0001. Red colored asterisks indicate fewer interactions with or less time spent with an intruder mouse in female *bArKO* or *tArKO* mice compared to littermate control or WT mice, respectively.

Social interaction testing was performed in old *bArKO* and *tArKO* mice of both sexes. Compared to sex‐matched littermate control mice, old female but not old male *bArKO* mice interacted fewer times and spent less time with a sex‐matched conspecific mouse (i.e., an intruder mouse) (Figure 4A,B), suggesting impaired social behavior during the social interaction test. Old female *tArKO* mice also interacted fewer times with the sex‐matched conspecific mouse; however, time spent with the conspecific was not different compared to WT females (Figure 4C,D). Social interaction time and number were not different in male *bArKO* and *tArKO* mice compared to littermate controls (Figure 4). As expected, control and WT littermates spent more time or had a greater number of interactions with the sex‐matched conspecific intruder mouse than the empty chamber without a mouse. These data suggest that aromatase deletion and estrogen deficiency in the brain affect social behavior in a sex‐specific manner, with impairment in old female but not old male *bArKO* or *tArKO* mice.

### 3.6 Anxiety‐Like Behavior and Locomotor Activity in *bArKO* and *tArKO* Mice

Locomotor activity was measured in young and old *bArKO* and *tArKO* mice of both sexes using the open field (OF) test (Figure S4). Only old female *tArKO* mice were significantly less active (measured as travel distance) compared to sex‐matched WT mice (Figure S4D). Locomotor activity tended to decrease but did not reach significance in old *tArKO* male mice (*p* = 0.05). In addition, locomotor activity was not different in *bArKO* mice by sex or age and in young *tArkO* mice of both sexes (Figure S4). Furthermore, the time spent in the center of the apparatus and the number of entries into the center for a measure of anxiety were similar between *bArKO* mice and sex‐matched control mice at young and old ages (Figure S5A,B). We also did not observe any difference in the time spent in the center of the apparatus between WT and *tArKO* mice of both sexes and at young and old ages. However, a significantly lower number of entries into the center of the arena was observed in old *tArKO* female mice but not young *tArKO* female mice and not male *tArKO* mice at young and old age as compared to littermate WT mice (Figure S5C,D). These results suggest greater anxiety‐like behavior in old female *tArKO* mice. Light–dark (LD) testing was used to further evaluate anxiety‐like behavior in *bArKO* and *tArKO* mice. However, no difference was found between any of the mice in the time spent or the number of entries into the light compartment (Figure S6). Overall, whole body aromatase knockout and decreased estrogen production in old *tArKO* mice were associated with sex differences in locomotor activities and possible anxiety‐like behavior.

### 3.7 Lower Brain Estrogen Is Associated With Alterations in Extracellular Matrix (ECM) Expression in the Hippocampus of Old Female *bArKO* Mice

**FIGURE 5:**
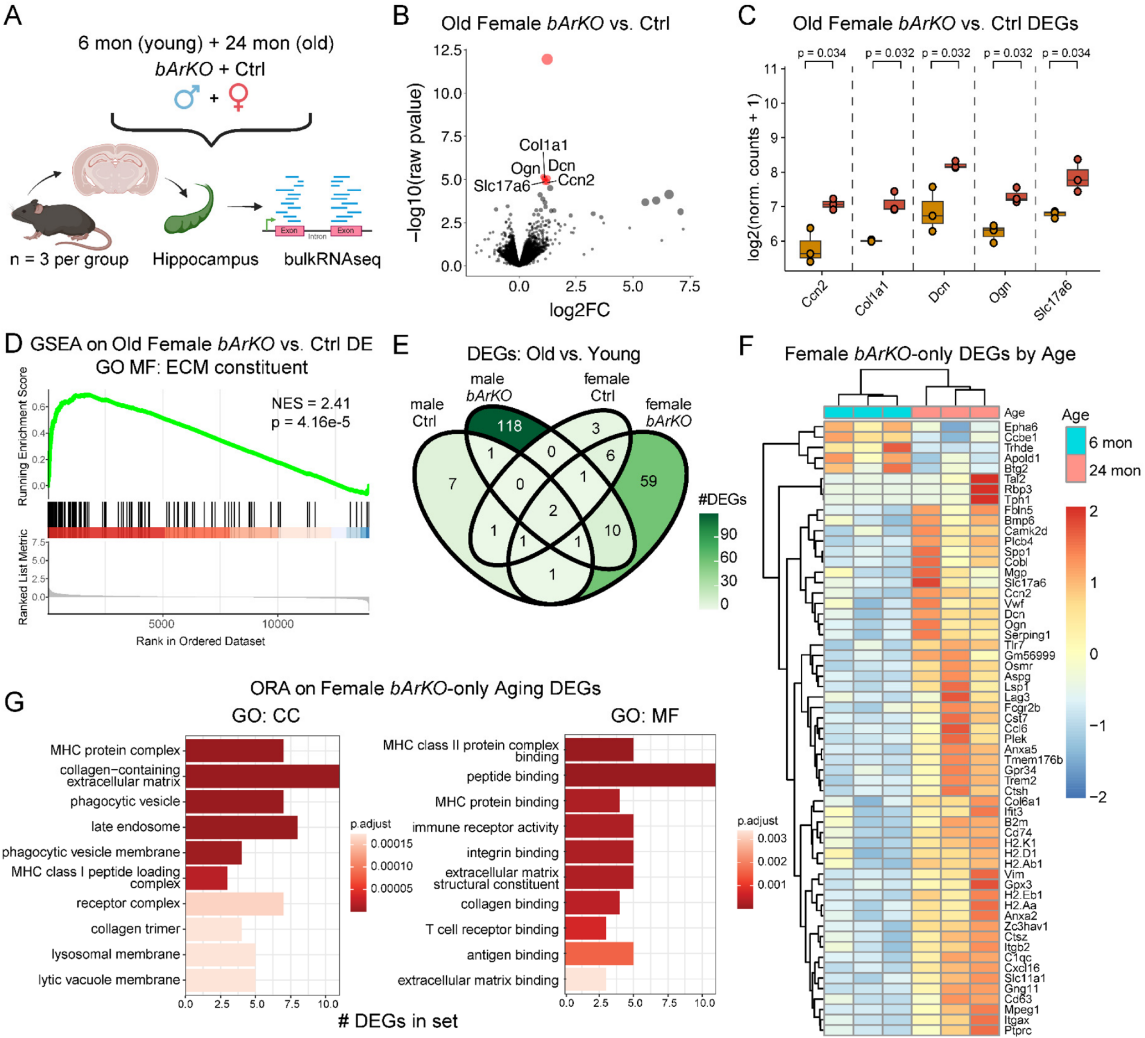
Bulk RNA‐seq analysis of hippocampi from young and old *bArKO* mice of both sexes demonstrates dysregulation of extracellular matrix constituents only in old *bArKO* females compared to age‐matched littermate controls (Ctrls). (A) Schematic of experimental design for bulk RNA‐seq analyses in *bArKO* vs. Ctrl mice across sex and age. (B) Volcano plot displaying differential expression (DE) between old female *bArKO* and Ctrl mice using Wald test. Dot sizes correspond to ‐log10(adjusted *p* value) multiplied by log‐fold change (LFC). Dots labeled in red passed the significance threshold (LFC > 0.50, adjusted *p* value < 0.05). (C) Boxplots showing differentially expressed genes (DEGs) between old female *bArKO* and Ctrl mice, counts normalized with median of ratios method. *p* values are calculated using Wald test and adjusted using Benjamini–Hochberg correction. (D) Gene set enrichment analysis (GSEA) of DEGs between old female *bArKO* and Ctrl mice using gene ontology (GO) molecular function (MF) sets. The most significantly enriched pathway (extracellular matrix [ECM] constituent) is shown. NES = normalized enrichment score. *p* value from permutation testing. (E) Venn diagram of DEGs across age in each sex and genotype combination. (F) Expression heatmap of genes differentially expressed between age groups of female *bArKO* mice only. Values are counts normalized using the median of ratios method, scaled across rows. (G) Pathway enrichment analysis (over‐representation analysis, ORA) of genes differentially expressed between age groups in female *bArKO* mice only using GO MF and cellular component (CC) sets. The top 10 most significantly enriched sets in old female *bArKO* mice per ontology are shown. *p* values are calculated using hypergeometric test.

To explore the underlying molecular mechanism for the observed sex‐specific memory loss and impaired behavior in *bArKO* mice, bulk RNA‐seq analysis of hippocampal tissue from young (6 months) and old (24 months) *bArKO* mice of both sexes was performed (*n* = 3/group, Figure 5A). We did not find any differentially expressed genes (DEGs) in young *bArKO* vs. sex‐matched control mice of both sexes (Figure S7). However, in old mice, we found 5 upregulated DEGs (*Ccn2, Col1a1*, *Dcn*, *Ogn*, and *Slc17a6*) in female *bArKO* mice and 2 upregulated DEGs (*Rreb1*, *Irx2*) in male *bArKO* mice compared to age‐ and sex‐matched controls (Figure 5B,C and Figure S8A,B). Gene set enrichment analysis (GSEA) was run using the captured gene list, ranked by log‐fold change between old female *bArKO* and old female control mice. The most significantly enriched gene ontology (GO) molecular function (MF) set in old female *bArKO* mice was extracellular matrix (ECM) constituents (Figure 5D). To further investigate how the effect of aging on hippocampal gene expression is altered by sex and aromatase status, we compared DEGs between old and young mice in each sex‐genotype group. All four sex‐genotype groups demonstrated upregulation of *C4b* and *Ighm* with age; both of these genes have been implicated previously in brain aging (Figure 5E). Intriguingly, 7 unique DEGs in old male control mice, 118 in old male *bArKO* mice, 3 in old female control mice, and 59 in old female *bArKO* mice were identified compared to their young counterparts (Figure 5E, Table S1). The 118 DEGs unique to old male *bArKO* mice were significantly enriched for the components of postsynaptic membrane, myelin sheath, and main axon sets when assessed by overrepresentation analysis using GO molecular function (MF) and cellular component (CC) sets (Figure S8C,D). In contrast, the 59 DEGs unique to old female *bArKO* vs. young female *bArKO* mice were not enriched for synaptic function‐related genes. Interestingly, the top 10 significantly overrepresented gene sets in old female *bArKO* mice DEGs included predominantly ECM (ECM structural constituent, ECM binding, collagen binding, collagen trimer, and integrin binding) and inflammation (MHC protein complex and binding, late endosome, lysosomal membrane, immune receptor activity, T cell receptor binding, and antigen binding) related pathways (Figure 5F,G). Overall, whole genome transcriptional analysis of *bArKO* and control hippocampus from young and old mice of both sexes showed sex‐, age‐, and estrogen loss‐specific gene expression patterns, with unique alterations of ECM component expression in old female mice with selective loss of brain estrogen (*bArKO*).

### 3.8 Validation of Differentially Expressed Genes in Female *bArKO* Mice

**FIGURE 6:**
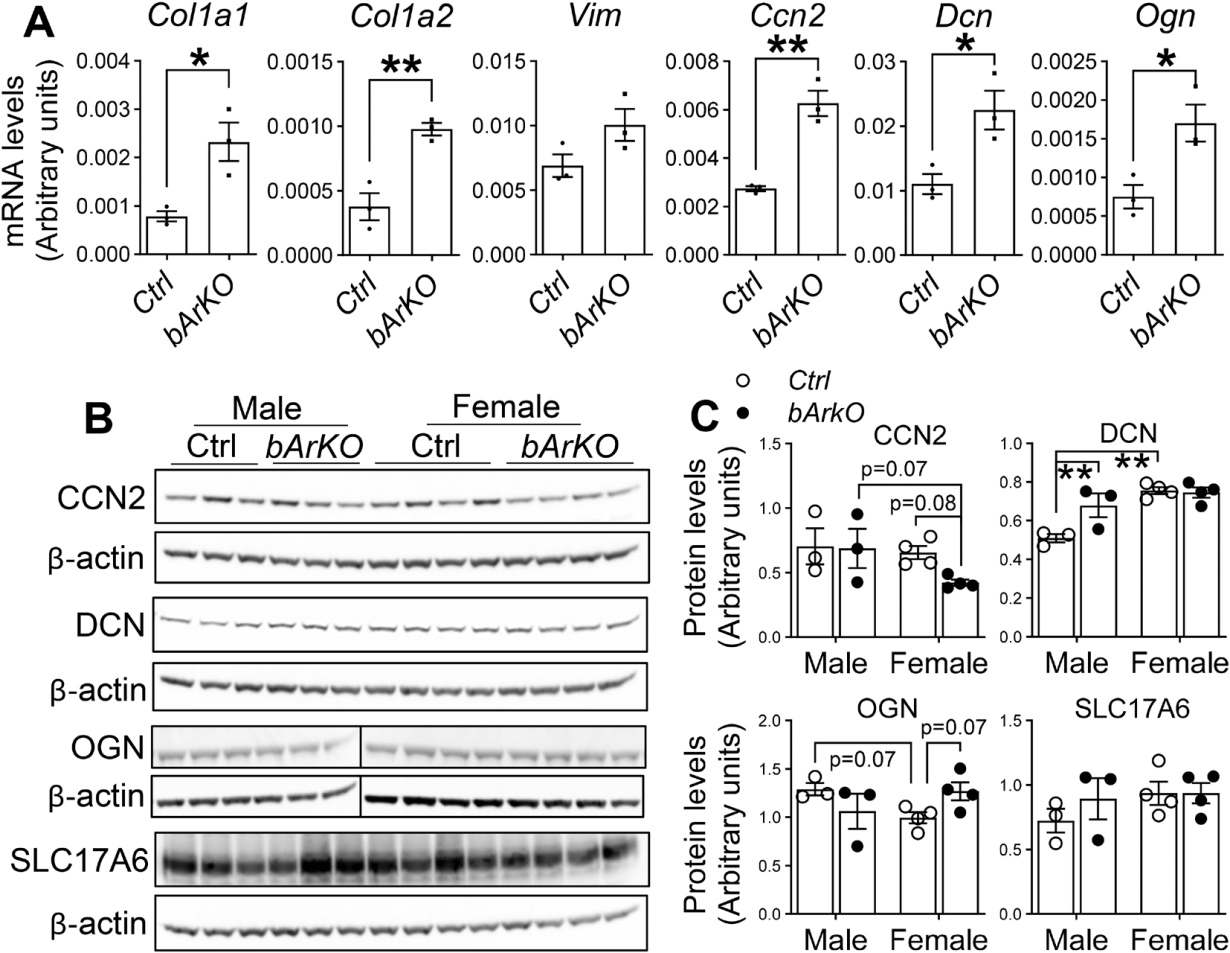
ECM gene expression in the hippocampus of old female *bArKO* mice of both sexes. (A) mRNA levels of ECM genes (*Col1a1*, *Col1a2*, *Vim*, *Ccn2*, *Dcn*, and *Ogn*) in old female *bArKO* vs. old female control (Ctrl) mice at the age of over 19–23 months. *Gapdh* mRNA levels served as loading controls. *n* = 3. Two‐tailed Student's *t*‐test was used. Protein expression (B) and quantification (C) of ECM components in the hippocampus of old (~20 months) *bArKO* mice of both sexes. β‐Actin served as a loading control. *n* = 3–4. Two‐way ANOVA with Tukey's multiple comparison test was used.

We validated the altered expression of ECM‐related genes (*Col1a1*, *Col1a2*, *Vim*, *Ccn2*, *Dcn*, and *Ogn*) and a vesicular glutamate transporter gene (*Slc17a6*) in old (~20 months) female *bArKO* mice (Escartin et al. 2021; Ito et al. 2016; Mann et al. 2017; Neo and Tang 2017; Schneider et al. 2021; Schwab et al. 2000). We found significantly higher expression of *Col1a1*, *Col1a2*, *Ccn2*, *Dcn*, and *Ogn* as well as nonsignificant but trending higher expression of *Vim* in the hippocampus of old female *bArKO* mice compared to old female control mice by qPCR (Figure 6A). We first measured protein levels of CCN2, DCN, and SLC17A6 in the hippocampus from female *bArKO*, *tArKO*, and WT control mice at old ages using immunofluorescence staining. The immunofluorescence density of CCN2, DCN, and SLC17A6 in the hippocampal CA1 region was similar in *bArKO* and *tArKO* mice as compared to control mice (Figure S9). We further used more sensitive immunoblotting to quantify these protein expressions. Protein levels of OGN were similarly higher (*p* = 0.07) in the hippocampus of old female *bArKO* compared to old female control mice, whereas CCN2 was lower (*p* = 0.08) (Figure 6B,C). Protein levels of DCN and SLC17A6 were similar across old *bArKO* and control female mice (Figure 6B,C). We thus found subtle differences in ECM‐related protein expression in the hippocampus of female *bArKO* mice as compared with control littermates. The subtlety of the observed differences and discordant RNA‐to‐protein levels for CCN2 expression can partially be explained by differential expression in distinct cell types, which are not captured in whole hippocampal lysates. TGFβ and Wnt signaling pathways are well‐established regulators of ECM remodeling. However, hippocampal mRNA levels of TGFβ ligands (*Tgfb1*, *Tgfb2*, *Tgfb3*), TGFβ receptors (*Tgfbr1*, *Tgfbr2*), and Wnt ligands (*Wnt1*, *Wnt3a*, *Wnt5a*) were comparable between old control and old *bArKO* mice in the bulk RNA‐seq dataset (Figure S10A,B). Consistently, protein levels of TGFBR2 and WNT1 were also similar between old female control/WT, *bArKO*, or *tArKO* mice (Figure S10C). Future single‐cell gene expression analyses of these and other ECM‐related genes and pathways may provide more meaningful information about the effect of age, sex, and estrogen signaling on the brain ECM signaling.

### 3.9 ER Expression in the Hippocampus of *bArKO* Mice

E2 binds primarily to three receptors (ERα, ERβ, and Gper1) to exert its biological functions in the brain (Hewitt and Korach 2018). To identify the estrogen receptors that mediate the observed effects of estrogen loss on memory and cognition, we measured ERα, ERβ, and Gper1 mRNA levels by qPCR in the hippocampus of young and old control and *bArKO* mice of both sexes (Figure S11). The hippocampus is associated with spatial working memory and social interaction; thus, ER signaling in this area is relevant to AD‐related cognitive impairment (Kalman and Keay 2017; Lalonde 2002; Rao et al. 2022). We found that hippocampal ER mRNA levels were not significantly different among any of the experimental groups (Figure S11A–C); however, ERα was expressed at higher levels than ERβ and Gper1 in the hippocampus of both *bArKO* and littermate controls at both ages. ERα mRNA levels were 6.3‐ to 16‐fold higher than ERβ and 3.3‐ to 5.2‐fold higher than Gper1 (Figure S11D,E). In addition, ERα protein levels were not different between old *bArKO* and control mice of both sexes (Figure S11F).

To further determine which estrogen receptors (ERα, ERβ, Gper1) in specific hippocampal cell types may mediate local brain estrogen loss‐induced ECM dysregulation, we reanalyzed single‐cell RNA‐seq data from the mouse hippocampus in the publicly available Allen Brain Cell (ABC) transcriptomic atlas (Yao et al. 2023), focusing on the expression of three estrogen receptors and ECM‐related genes (*Col1a1, Ccn2, Dcn, Ogn*). Eight broader cell type groups were identified (glutamatergic, GABAergic, and dentate gyrus neurons, oligodendrocytes, immune cells [microglia], astrocytes, vascular cells, and Cajal–Retzius cells) (Figure S12A). *ERα* was highly expressed in neurons and immune cells, followed by astrocytes, oligodendrocytes, and vascular cells. Except for relatively high *Gper1* expression in vascular cells, *ERα* expression was higher than *ERβ* and *Gper1* expression across hippocampal cell types (Figure S12B). ECM‐related genes also exhibited distinct cell type‐specific patterns, with *Col1a1* enriched in astrocytes; *Dcn* in glutamatergic and dentate gyrus neurons; *Ogn* in dentate gyrus neurons; and *Ccn2* primarily in vascular cells, with subsets of vascular cells additionally expressing *Col1a1*, *Dcn*, and *Ogn* (Figure S12C). In addition, selective deletion of ERβ in astrocytes, but not neurons, in gonadally intact female mice induces hippocampal‐dependent cognitive impairment, dorsal hippocampal atrophy, and astrocyte and microglial activation with synaptic loss, while transcriptomic analyses of hippocampal astrocytes from the astrocyte‐specific ERβ deletion mice indicate that metabolic pathways (e.g., gluconeogenesis and glycolysis), rather than ECM pathways, are most affected (Itoh et al. 2023); our reanalysis of this dataset confirmed that ECM gene expression (*Col1a1*, *Dcn*, *Ogn*, *Ccn2*) remained largely unchanged (Figure S13). Taken together, these data suggest that astrocytes, glutamatergic and dentate gyrus neurons, and vascular cells are the major cell types involved in ECM dysregulation, with ERα likely playing a central role due to its high expression across these cell types. In contrast, other estrogen receptors may regulate hippocampal function through distinct, non‐ECM mechanisms.

## 4 Discussion

In rodents, aromatase is expressed almost exclusively in the brain and gonads (Bulun et al. 2005; Zhao et al. 2009). Old female mice are thus heavily reliant on estrogen synthesis in the brain after the cessation of estrous cycles (equivalent to menopause in women). The characteristics of estrogen synthesis motivated us to generate a unique *bArKO* transgenic mouse model to study estrogen's impact on brain function, and the effect of estrogen deficiency in the aging brain after reproductive senescence (i.e., menopause in humans), which may contribute to sex differences in AD vulnerability. In our study, old female *bArKO* mice exhibited sex‐ and age‐specific memory loss and reduced social interaction compared to old male *bArKO* mice; these differences were associated with brain‐selective aromatase deletion leading to estrogen deficiency in the brain. Most interestingly, RNA‐seq analysis revealed enhanced expression of ECM‐related pathway components and ECM‐related gene dysregulation in old female *bArKO* mice compared to old littermate control mice. Thus, *bArKO* mice represent a unique and physiologically relevant model to test the essential role of estrogen in brain function and the sex‐specific impact of brain estrogen deficiency during aging on memory, affective behavior, and gene expression profiles. We included *tArKO* mice in our study to comprehensively assess the contributions of brain vs. systemic estrogen loss to observed sex differences in AD vulnerability. This study advances our understanding of the molecular consequences of the loss of estrogen action in the brain and potential mechanisms driving higher AD vulnerability in women.

Estrogens, essential regulators of female reproductive function and secondary sex characteristics, broadly modulate neuronal function by binding to multiple membrane and nuclear receptors expressed throughout the brain (Hara et al. 2015). Fluctuations in estrogen levels shape emotional state and cognitive performance in women across the lifespan, such that estrogen deficiency increases vulnerability to mood and memory disorders in both humans and rodent models (Shepherd 2001; Wharton et al. 2012). Ovariectomy and estrogen synthesis inhibition in mice trigger significant neurophysiological changes, primarily impaired memory, increased anxiety/depression‐like behaviors, reduced synaptic plasticity, and dendritic spine loss, particularly in the hippocampus and cortex (Baek et al. 2024; Liu et al. 2024; Tao et al. 2020; Tuscher et al. 2016). Evidence from rodent studies confirms that blocking estrogen synthesis by pharmacologically inhibiting or whole‐body genetic deletion of the aromatase enzyme leads to significant negative impacts on the brain, particularly in the hippocampus (Brann et al. 2021). Both systemic (e.g., letrozole) and local (intrahippocampal) inhibition of aromatase lead to deficits in hippocampus‐dependent memory, such as spatial reference memory and object recognition (Bayer et al. 2015; Tuscher et al. 2016). Consistent with pharmacological inhibition of estrogen synthesis, whole‐body aromatase knockout (*ArKO*) mice also exhibited impaired spatial working memory (Martin et al. 2003). Inhibition of aromatase reduces hippocampal spine synapse density, especially in females (Zhou et al. 2010), and impairs long‐term potentiation in the hippocampus, which is the cellular basis for learning and memory (Vierk et al. 2012). Therefore, the lack of estrogen‐whether from surgery or synthesis blockage‐removes crucial neuroprotective and plasticity‐enhancing signals, leading to brain aging‐related symptoms, cognitive deficits, and emotional instability.

Several mouse models, including *ArKO* mice, have been used to investigate the impact of estrogen deficiency on memory and Aβ accumulation, but none have compared the sex‐ or age‐dependent effects of brain‐specific estrogen loss, from memory and behavioral effects to changes in gene expression profiles (Bayer et al. 2015; Overk et al. 2012; Tuscher et al. 2016; Yue et al. 2005). *tArKO* mice, possessing the same genetic background as *bArKO* mice, were used as positive controls due to their severe systemic loss of estrogen, in contrast to the brain‐specific estrogen deficiency in *bArKO* mice. Our group is the first to generate this genetically modified *bArKO* mouse model, enabling us to investigate the impact of brain‐specific estrogen deficiency on the memory function and affective behavior before and after reproductive senescence, mimicking menopause in humans. Young *bArKO* mice maintain normal systemic levels of E2 up to 12 months of age (equivalent to perimenopause in women); therefore, E2 continues to circulate to the brain to compensate for the loss of local aromatase deletion and estrogen deficiency. However, loss of systemic hormones in old *bArKO* mice, after reproductive senescence, leads to a decrease in E2 levels in the brain, mimicking the estrogen deficiency seen in postmenopausal women with AD (Ishunina et al. 2007; Li et al. 2014). We also demonstrated that only old female *bArKO* mice showed impaired working memory and affective behaviors, further supporting the importance of estrogen in maintaining brain function and protecting against AD in women.

AD is characterized as memory loss that often presents with a variety of neuropsychiatric symptoms; affective behavior is one of the most common neuropsychiatric symptoms in AD (Fisher, Dunn, Keszycki, et al. 2024; Matan‐Lithwick et al. 2025). There are several types of memory functions in humans that can be tested in rodent models, such as short‐ and long‐term memory, special working memory, and recognition memory. In this study, the Y‐maze test was used to assess short‐term spatial working memory; novel objective and social recognition memory were tested by NOR and SR tests in *bArKO* and *tArKO* mice, respectively. We found that old *bArKO* mice had impaired working memory, but recognition memory and social memory were not different, indicating that brain aromatase deletion and estrogen loss may specifically impact short‐term spatial working memory. This is consistent with previous findings in women, which showed that estrogen loss impairs working memory in postmenopausal women or in young women given estrogen synthesis inhibitor or selective estrogen receptor modulator (e.g., tamoxifen) (Hampson 2018).

Disordered affective behavior, referring to the dysregulation of emotional behavior, is one of the most common neuropsychiatric symptoms of AD (Fisher, Dunn, and Dong 2024; Fisher, Dunn, Keszycki, et al. 2024; Matan‐Lithwick et al. 2025). Affective behavior disorders, particularly those related to depression and anxiety, can emerge in early stages of AD, such as mild cognitive impairment, and are linked to increased risk and potentially accelerated progression of AD (Burke et al. 2018; Byers and Yaffe 2011; Elser et al. 2023). Recent studies have also suggested that anxiety increases the risk for AD independent of the presence of depression (Mendez 2021; Santabarbara, Lopez‐Anton, et al. 2019; Santabarbara, Villagrasa, et al. 2019). Further, fluctuations in estrogen levels, particularly during times of hormonal change such as puberty, menstruation, postpartum, and perimenopause, can significantly impact mood and increase vulnerability to depression (Q. Sun et al. 2024). The significant drop in estrogen during perimenopause and menopause can increase susceptibility to depression and anxiety (Wada et al. 2018), which are twice as common in women compared to men. We found significantly greater depressive‐like behavior and possible anxiety behavior only in old female *tArKO* mice; however, the mechanisms underlying this gender difference in the effect of estrogen loss are unclear. Further, depressive‐like behavior occurred exclusively in female *tArKO* mice, but not in female *bArKO* mice, suggesting that severe estrogen deficiency in the whole body (in both circulation and brain) contributes to this difference in female mice. Brain‐specific estrogen loss did not induce depressive‐like behavior in both male and female mice at young and old ages. Consistent with our observations, depressive‐like behavior assessed by the forced swim test was detected exclusively in ovariectomized, but not intact, female forebrain neuron–specific aromatase knockout mice (Lu et al. 2019). The mechanisms by which systemic versus local brain estrogen deficiency influences depression remain unclear; however, previous studies have shown that brain‐derived estrogen functions as a neuromodulator and is critical for hippocampal‐dependent memory, synaptic plasticity, and neuroprotection against injury (Brann et al. 2022; Kretz et al. 2004; Taxier et al. 2020).

Another important finding in this study is that enhanced pathways and DEGs associated with ECM (e.g., *Col1a1, Ccn2, Dcn, and Ogn*) were identified in old female *bArKO* mice. Previous studies indicated that E2 can induce ECM accumulation and aberrant ECM remodeling (i.e., fibrosis) in various diseases, including inguinal hernia, gynecomastia, scleroderma, and uterine fibroid tumors (Agarwal et al. 1998; Aida‐Yasuoka et al. 2013; Luo et al. 2014; Potluri et al. 2025; Zhao et al. 2018). The ECM in the brain is primarily composed of glycosaminoglycans (e.g., hyaluronan), proteoglycans (e.g., neurocan, brevican, versican and aggrecan), glycoproteins (e.g., tenascin‐R), and fibrous proteins (e.g., collagen, fibronectin, and vitronectin) (Burnside and Bradbury 2014; Dityatev, Schachner, and Sonderegger 2010; Song and Dityatev 2018), which account for approximately 20% of the total volume of the brain (Nicholson and Sykova 1998). ECM is synthesized and excreted by both neurons and glial cells to provide neural cells with anchor points and facilitate the organization of these cells into distinct central nervous system regions (Dityatev, Seidenbecher, and Schachner 2010). Additionally, studies have shown that ECM regulates several fundamental neural processes during brain development, including neurite outgrowth, synaptogenesis, and synaptic stabilization (Burnside and Bradbury 2014; Song and Dityatev 2018). ECM molecules can also regulate various aspects of synaptic plasticity and prevent abnormal synaptic remodeling, which is integral to learning and memory (Dityatev and Schachner 2003; Dityatev, Schachner, and Sonderegger 2010; Lau et al. 2013). ECM alterations, including degradation, overproduction, and altered composition, have been identified in neurodegenerative diseases such as AD, Huntington's disease, and Parkinson's disease (Bonneh‐Barkay and Wiley 2009; Pinter and Alpar 2022; Y. Sun et al. 2021). Despite the well‐established role of estrogen in regulating the ECM of other tissues and the significance of ECM alterations in neurodegeneration, little attention has been focused on this connection in explaining sex‐based differences in AD vulnerability. In this study, gene set enrichment analysis showed aromatase deletion and estrogen deficiency were associated with ECM alterations in the hippocampus of old female (but not male) *bArKO* mice. Interestingly, ECM‐related pathways (e.g., ECM structural constituent, ECM binding, collagen binding, collagen trimer, and integrin binding) were in the top 10 significantly enriched pathways in the hippocampus from old female *bArKO* mice. Four of five upregulated DEGs (*Col1a1*, *Ccn2*, *Dcn*, and *Ogn*) in female *bArKO* mice were either ECM‐related genes or interacted with ECM‐related genes (Fernandez‐Medarde et al. 2007; Nulali et al. 2022; Y. Shi et al. 2022; Soles et al. 2023; Yang et al. 2017). Given the low levels of collagen fibrils in the brain, even a mild alteration to this aspect of the ECM could be significant. The known roles of DCN and OGN in collagen binding and CCN2 in upregulation of Collagen I production further support the notion of an altered collagen environment in the estrogen‐deficient brain (Chen et al. 2020; Lin et al. 2013; Nulali et al. 2022). Our study suggests a potential link between postmenopausal estrogen loss‐driven alterations in ECM and memory and affective behaviors in mice. In humans, excess ECM accumulation may contribute to AD pathology (Amontree et al. 2023; C. Shi et al. 2023). mRNA levels of ECM‐related genes (*COL1A1, CCN2, DCN, OGN*) are significantly elevated in the hippocampus of patients with AD compared to age‐matched cognitively normal controls (van Rooij et al. 2019). Consistently, collagen protein levels (e.g., COL1A1) are differentially expressed in the AD brain and strongly correlate with disease status (C. Shi et al. 2023). CCN2 protein is detected predominantly in neurons in old cognitively normal controls, but is present in both neurons and astrocytes in age‐matched patients with AD (Ueberham et al. 2003). Furthermore, its expression in the brain positively correlates with clinical disease progression and Aβ neuritic plaque burden (Zhao et al. 2005). In cerebrospinal fluid (CSF), DCN levels are positively correlated with Aβ42, suggesting an association with early Aβ amyloidosis (Jiang et al. 2022; Tijms et al. 2020). However, these findings are derived from studies of mixed‐sex cohorts of AD patients and age‐matched controls. Future studies are needed to determine how sex influences ECM gene and protein expression in AD, particularly in postmenopausal women compared to age‐matched men and cognitively normal individuals.

Estrogen binding to and activating ER has been implicated in the regulation of hippocampal function, including spatial learning and memory (Spencer et al. 2008). Estrogen induces changes in gene expression (classical genomic effects) via ERα and ERβ or rapid activation of membrane‐associated signaling pathways (nongenomic effects), mainly via GPER1. ERs are present in various hippocampal cell types, including neurons, astrocytes, and microglia (Baek et al. 2024; Goenaga et al. 2025; McEwen et al. 2001; Sierra et al. 2008). We found that ER expression in the hippocampus was unaffected by sex, aging, or estrogen deficiency. Previous studies showed that ERα or ERβ expression is modulated by estrogen levels and decreases in distinct structures (e.g., CA1 region) or cellular compartments (e.g., synapse/spines) of the hippocampus during aging (Bean et al. 2014; Hara et al. 2015; Mehra et al. 2005; Waters et al. 2011). However, minimal literature is available on how ER expression changes in the hippocampus of aging humans. ER protein is higher in the dentate gyrus and CA3 region but lower in the CA1 region of the hippocampus in aged humans (Tohgi et al. 1995). In contrast, GPER1 expression was shown to be unaffected by aging and surgical menopause (Prossnitz and Barton 2011). Surprisingly, a recent in vivo brain ^18^F‐fluoroestradiol positron emission tomography study of healthy midlife women reveals progressively higher ER density over the menopause transition (Mosconi et al. 2024). These contrasting reports motivate the need for future studies on region‐, location‐, and cell type‐specific ER expression in the brain that may provide additional insights into ER functional differences across sex, age, and estrogen status. Previous studies have shown that ERα plays a prominent neuroprotective role (Spence et al. 2011, 2013). Additionally, restoration of ERα expression in hippocampal neurons of ERα knockout mice rescues estrogen responsiveness and spatial learning (Foster et al. 2008); however, whether this rescue is mediated through ECM pathways remains unclear. In addition, ERβ in astrocytes, but not neurons, regulates hippocampal function through distinct, non‐ECM mechanisms (Itoh et al. 2023). In the future, we will selectively delete ERα in neurons, astrocytes, or microglia using stereotactic AAV‐Cre delivery to the hippocampus to determine whether cell‐type‐specific ERα mediates brain estrogen loss‐induced ECM dysregulation.

Although we defined the role of estrogen in sex‐specific changes in memory, affective behavior, and hippocampal ECM gene expression, we are aware of several limitations in this study. First, small sample sizes were used in certain experiments. In behavioral tests, we initially aged ~15 mice per group for behavioral testing at an advanced age (> 19 months old). The full behavioral testing series used in this study required approximately 1.5 months to complete. During the aging process and behavioral testing period, some mice died. In addition, a subset of mice was excluded from specific behavioral assays due to inappropriate performance (e.g., failure to move during the test). To minimize experimental variability and avoid potential learning or carryover effects, each behavioral test was conducted only once per mouse. Consequently, sample sizes varied among experimental groups and across different behavioral assays, with some groups having relatively small sample sizes in certain tests. In addition, we used 3 mice per group in bulk RNA‐seq and qPCR, as well as 3 to 4 mice per group in immunoblotting validation. While acceptable in exploratory studies, a small number in the research groups may reduce statistical power, making it difficult to draw firm conclusions from near‐significant trends (e.g., protein‐level *p* = 0.07 or 0.08). In addition, while ECM‐related gene expression changes were evident at the mRNA level, the corresponding changes at the protein level were inconsistent, with some not reaching statistical significance. Second, our study found the correlation between upregulation of hippocampal ECM‐related genes and memory and behavioral deficits in old female *bArKO* mice. However, it is not clear whether ECM alterations directly cause cognitive impairment. In the future, we will pharmacologically or genetically target these upregulated ECM components (e.g., Col1a1, Dcn) and assess whether cognitive deficits can be reversed. Conversely, we will overexpress these ECM genes in aged wild‐type female mice to determine if they recapitulate similar cognitive impairments. Third, bulk RNA‐seq analysis was used for identifying overall DEGs between control and *bArKO* mice of both sexes. The hippocampus is a heterogeneous tissue with various cell types. It is impossible to define cell type‐specific DEGs between control and *bArKO* mice. We plan to perform single‐nucleus RNA‐seq to determine which cell types contribute to changes in hippocampal ECM‐related genes between control and *bArKO* mice. Lastly, we assessed memory in *bArKO* mice using the Y‐maze (spatial working memory), novel object recognition, and social recognition tests. Only aged female *bArKO* mice exhibited deficits in the Y‐maze. However, as AD is characterized by progressive impairment in hippocampal‐dependent long‐term episodic memory, the lack of assays such as the Morris water maze or contextual fear conditioning limits the interpretation of these findings in the context of canonical AD‐related cognitive decline. These limitations will be addressed, where possible, in future studies.

## 5 Conclusions

**FIGURE 7:**
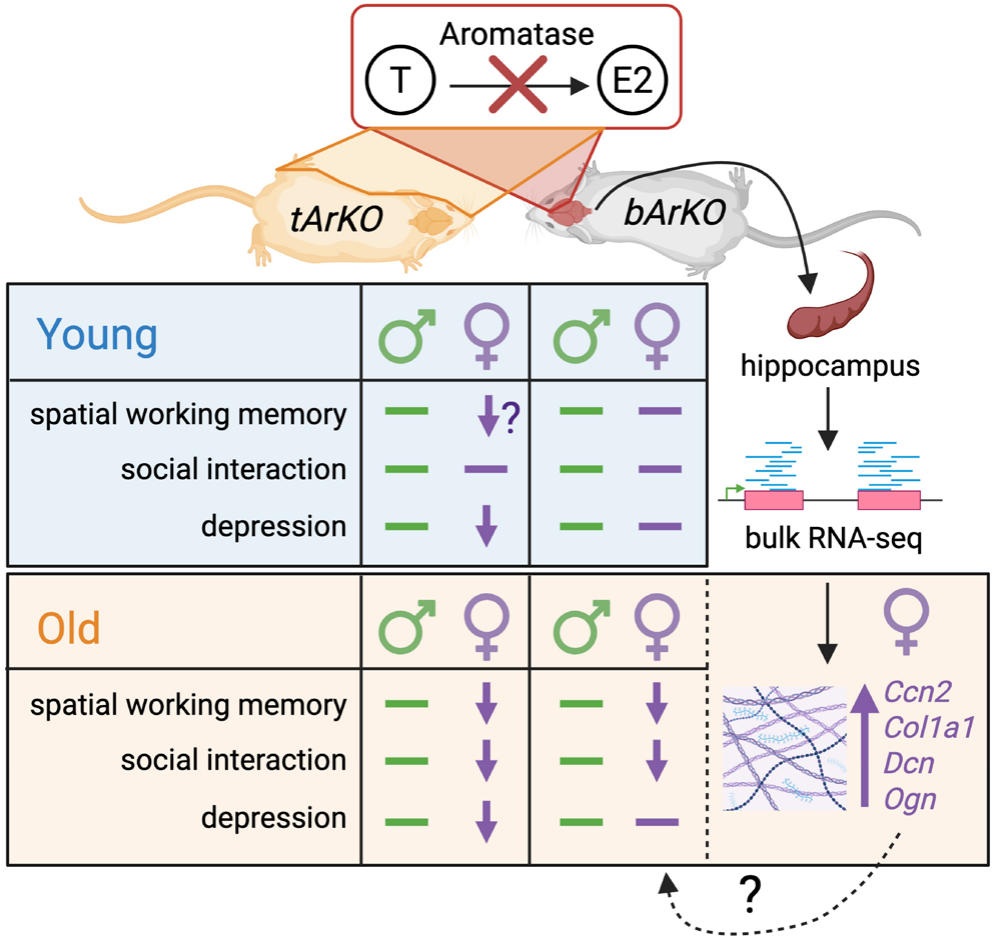
Schematics showing the association between estrogen deficiency, memory loss, and ECM dysregulation in the brain. Brain‐specific aromatase knockout (*bArKO, right*) mice exhibited spatial working memory, social interaction impairment, and hippocampal ECM dysregulation in old female mice but not in old male or young *bArKO* mice of either sex. In addition to the same behavior impairment in *bArKO* mice, whole body total aromatase knockout (*tArKO, left*) mice also presented depression in young and old female *tArKO* mice. ♂, males and ♀, females. Created with BioRender (biorender.com).

In summary, we used a unique animal model to reveal the impact of brain‐specific estrogen signaling on cognitive and behavioral endpoints. We revealed that estrogen regulates ECM gene expression in the hippocampus in a sex‐specific manner. Furthermore, brain‐specific estrogen deficiency, achieved through targeted deletion of aromatase, led to alterations in hippocampal ECM that correlated with behavioral changes and memory impairment. Whole‐body aromatase deletion, resulting in severe estrogen deprivation, is associated with robust behavioral abnormalities (Figure 7). In future studies, we will continue investigating whether manipulating ECM and other ER target genes can rescue memory deficits, affective behavioral changes, and neuropathology in an old AD mouse model with brain‐specific aromatase knockout as a model of vulnerability to AD after menopause.

## Author Contributions

N.C.P., A.W.H., G.R., A.L., S.K., C.H., R.P.M., J.C.V., Z.A.K., and T.Y. performed experiments or interpreted data. N.C.P., H.Z., A.W.H., G.R., and A.L. analyzed data. H.Z., N.C.P., S.E.B., and H.D. drafted the work or substantively revised it. H.Z., S.E.B., and H.D. conceived and supervised the study. All authors read and approved the final manuscript.

## Funding

This work was supported by the National Institutes of Health, RF1‐AG079419.

## Conflicts of Interest

The authors declare no conflicts of interest.

## Supporting information


**Figure S1:** Characterization of fertility and body weight in *bArKO* and *tArKO* female mice. Average litter size of (A) *bArKO* and (B) *tArKO* mice. The average number of pups per litter was calculated when control, *bArKO*, WT, or *tArKO* females were mated with age‐matched WT males for 4 months. Age‐matched floxed aromatase female mice were used as controls for *bArKO* mice and wild type (WT) mice served as controls for *tArKO* mice. two‐tailed Student's *t* test, *p < 0.05, *n* = 5 per group. Body weight was assessed weekly from 3 to 48 weeks of age in (C) female *bArKO* and (D) female *tArKO* mice. 2‐tailed Student's *t* test, *p < 0.05, *n* = 7–8 for controls, *n* = 8 for *bArKO* mice, *n* = 7 for WT and *tArKO* mice.
**Figure S2:** Memory for novel object recognition is not different among old *bArKO* and *tArKO* mice of both sexes. Novel object recognition (NOR) testing was performed in old *bArKO* mice (A and C) and old *tArKO* mice (B and D) of both sexes at the age of > 19 months. Discrimination index for time exploring the novel object was calculated for old *bArKO* (A) and *tArKO* (B) mice. Discrimination index for interactions with the novel object was calculated for old *bArKO* (C) and *tArKO* (D) mice. The number of mice used in the test is indicated in the graph. Two‐way ANOVA with Tukey's multiple comparison test was used.
**Figure S3:** Memory for social recognition is not different among old *bArKO* and *tArKO* mice of both sexes. Social recognition (SR) testing was performed in old *bArKO* mice (A and C) and old *tArKO* mice (B and D) of both sexes at the age of > 19 months. The experimental mouse was videotaped for 10 min; SR was measured by (A and B) the number of interactions with as well as (C and D) the time spent around the “old” and “new” intruder mice. For *bArKO* mice, *n* = 9 for control males, *n* = 5 for *bArKO* males, *n* = 6 control females, and *n* = 5 *bArKO* females. For *tArKO* mice, *n* = 8 for males and WT females and *n* = 6 for *tArKO* female mice. Two‐way ANOVA with Tukey's multiple comparison test was used.
**Figure S4:** Whole body aromatase knockout and decreased estrogen production are associated with low locomotor activity (LA) in old female *tArKO* mice. LA was measured in (A) young and (B) old *bArKO* mice as well as (C) young and (D) old *tArKO* mice. The number of mice used in the test is indicated in each column. Two‐way ANOVA with Tukey's multiple comparison test was used, *p < 0.05, **p < 0.01, ****p < 0.0001.
**Figure S5:** Whole‐body total aromatase knockout may link to anxiety‐like behavior in old female *tArKO* mice. The open field test (OF) was performed in young and old *bArKO* mice (A and B) and young and old *tArKO* mice (C and D) of both sexes. Less time spent in and fewer entries into the center of the apparatus indicate anxiety‐like behavior. Old female *tArKO* mice showed fewer entries into the center of the apparatus than WT mice. The number of mice used in the test is indicated in each column. Two‐way ANOVA with Tukey's multiple comparison test was used. *p < 0.05.
**Figure S6:** *bArKO* and *tArKO* mice do not exhibit anxiety‐like behavior in the light/dark box test. The light–dark box test (LD) was performed in old *bArKO* and old *tArKO* mice of both sexes at age > 19 months. We measured time in the light compartment of the box (A, C) and entries into the light compartment (B, D) for *bArKO* mice (A, B) and *tArKO* mice (C, D). There was no difference between all groups for time spent in or the number of entries into the light compartment of the box. Mice spent less time and had fewer entries into the light compartment of the box suggesting higher levels of anxiety‐like behavior. The number of mice used in the test is indicated above each column in the graph. Two‐way ANOVA with Tukey's multiple comparison test was used.
**Figure S7:** Bulk RNA‐seq analysis of hippocampi shows no significant dysregulation between young *bArKO* and littermate control (Ctrl) mice of both sexes. Volcano plot showing differential gene expression between (A) young male *bArKO* and (B) young female *bArKO* and their sex‐matched littermate control mice using Wald test.
**Figure S8:** Bulk RNA‐seq analysis of hippocampi from *bArKO* male mice across age. (A) Volcano plot displaying differential expression (DE) between old male *bArKO* and littermate control (Ctrl) mice using Wald test. (B) Boxplots showing differentially expressed genes (DEGs) between old male *bArKO* and Ctrl mice; counts normalized using the median of ratios method. *p* values are calculated using Wald test and adjusted using Benjamini–Hochberg correction. (C) Pathway enrichment analysis (over‐representation analysis) of DEGs by age for male *bArKO* mice only using GO MF and cellular component (CC) sets. Up to the top 10 most significantly enriched sets per ontology are shown. *p* values are calculated using hypergeometric test. (D) Expression heatmap of DEGs by age in male *bArKO* mice only. Values are counts normalized using the median of ratios method, scaled across rows.
**Figure S9:** Validation of ECM‐related proteins and SLC17A6 in *bArKO* and *tArKO* mice by immunofluorescent staining. (A‐C) Immunofluorescence staining was performed to assess the expression of ECM‐related proteins (CCN2 [A], DCN [B]) and SLC17A6 (C) in the brains of aged *bArKO* and *tArKO* mice (*n* = 3–4 per group). Co‐immunostaining with GFAP and NeuN was conducted to identify astrocytes and neurons, respectively. IgG immunostaining served as a negative control. (D‐F) Quantification of immunofluorescence signal intensity of CCN2 [D], DCN [E], and SLC17A6 (F) in the hippocampal CA1 region in control (Ctrl)/WT, *bArKO*, and *tArKO* mice. Kruskal–Wallis with Dunn's multiple comparisons test was used. Scale bars, 100 μM.
**Figure S10:** Major ECM regulating pathways, TGFβ and Wnt, are not significantly altered in the hippocampus of old female *bArKO* and *tArKO* mice. (A) Normalized RNA counts of TGFβ and Wnt ligands between aged female control (Ctrl) and *bArKO* hippocampus by bulk RNA‐seq. (B) Normalized RNA counts of TGFβ receptors 1 and 2 (*Tgfbr1*, *Tgfbr2*) between aged female Ctrl and *bArKO* hippocampus by bulk RNA‐seq. Immunoblotting showed expression (left) and quantification (right) of TGFBR2 and WNT1 in the hippocampus of *bArKO* (C) *and tArKO* (D) female mice at old age. β‐actin served as the loading control. *n* = 3–4. WT, wild type. The Mann–Whitney test was used.
**Figure S11:** ER expression in the hippocampus of *bArKO* mice. mRNA levels of *ERα* (A), *ERβ* (B), and *Gper1* (C) in the hippocampus of young (6 months) and old (> 19 months) *bArKO* mice of both sexes. Ratios of ERα/ERβ (D) and ERα/Gper1 (E) are calculated. *Gapdh* mRNA level served as the loading control. *n* = 3. Two‐way ANOVA with Tukey's multiple comparison test. (F) Immunoblotting showed ERα expression (top) and quantification (bottom) in the hippocampus of old *bArKO* mice of both sexes (~20 months). β‐actin served as the loading control. *n* = 3–4. ♂, males and ♀, females.
**Figure S12:** Hippocampal cell‐type specific expression of estrogen receptors and differentially expressed ECM genes identified in the mouse hippocampus in the Allen Brain Cell (ABC) transcriptomic atlas. (A) UMAP of 176 k hippocampus cells from adult mouse brain from the ABC atlas, colored by broad cell type. (B,C) UMAPs of cells from (A) colored for estrogen receptors (B) and differentially expressed ECM genes (C).
**Figure S13:** Differentially expressed ECM genes from the hippocampal astrocytes are not significantly altered between wild‐type and astrocyte ERβ conditional knockout (*ERβ cKO*) mice (GSE220288). (A) Schematic of experimental design. At 12 months of age, the hippocampus was collected for bulk RNA‐seq from wild‐type (WT) and *ERβ cKO* mice. (B) Normalized RNA counts of differentially expressed ECM genes (*Ccn2*, *Col1a1*, *Dcn*, *Ogn*) between WT and ERβ cKO female hippocampus. All comparisons are not significant (*p* > 0.05). Wald test through DESeq2 was used.

**Table S1:** DEGs between old and young mice in each sex‐genotype group (male/female‐control).

## Data Availability

The bulk RNA‐seq data from this study have been deposited in the NCBI Gene Expression Omnibus (GEO) database under the accession number GSE285197 (https://www.ncbi.nlm.nih.gov/geo).
