# Supplementary material for: Loss of Brain‐Derived Estrogen Is Associated With Sex‐ and Age‐Dependent Alterations in Memory, Affective Behavior, and Hippocampal Extracellular Matrix Gene Expression
Source: Aging Cell. 2026 May 26;25(6):e70551. doi: 10.1111/acel.70551 (PMC13239850; doi:10.1111/acel.70551)
Supplement: Supplementary file 1 — Figure S1: Characterization of fertility and body weight in bArKO and tArKO female mice. Average litter size of (A) bArKO and (B) tArKO mice. The average number of pups per litter was calculated when control, bArKO, WT, or tArKO females were mated with age‐matched WT males for 4 months. Age‐matched floxed aromatase female mice were used as controls for bArKO mice and wild type (WT) mice served as controls for tArKO mice. two‐tailed Student's t test, *p < 0.05, n = 5 per group. Body weight was assessed weekly from 3 to 48 weeks of age in (C) female bArKO and (D) female tArKO mice. 2‐tailed Student's t test, *p < 0.05, n = 7–8 for controls, n = 8 for bArKO mice, n = 7 for WT and tArKO mice. Figure S2: Memory for novel object recognition is not different among old bArKO and tArKO mice of both sexes. Novel object recognition (NOR) testing was performed in old bArKO mice (A and C) and old tArKO mice (B and D) of both sexes at the age of > 19 months. Discrimination index for time exploring the novel object was calculated for old bArKO (A) and tArKO (B) mice. Discrimination index for interactions with the novel object was calculated for old bArKO (C) and tArKO (D) mice. The number of mice used in the test is indicated in the graph. Two‐way ANOVA with Tukey's multiple comparison test was used. Figure S3: Memory for social recognition is not different among old bArKO and tArKO mice of both sexes. Social recognition (SR) testing was performed in old bArKO mice (A and C) and old tArKO mice (B and D) of both sexes at the age of > 19 months. The experimental mouse was videotaped for 10 min; SR was measured by (A and B) the number of interactions with as well as (C and D) the time spent around the “old” and “new” intruder mice. For bArKO mice, n = 9 for control males, n = 5 for bArKO males, n = 6 control females, and n = 5 bArKO females. For tArKO mice, n = 8 for males and WT females and n = 6 for tArKO female mice. Two‐way ANOVA with Tukey's multiple comparison test was used. Fi [file ACEL-25-e70551-s001.zip › ACEL_70551__pe supplemental figure legends 5.25.2026.docx]

**Supplemental Figure Legends**

**Figure S1 Characterization of fertility and body weight in *bArKO* and *tArKO* female mice.** Average litter size of (**A**) *bArKO* and (**B**) *tArKO* mice. The average number of pups per litter was calculated when control, *bArKO*, WT, or *tArKO* females were mated with age-matched WT males for 4 months. Age-matched floxed aromatase female mice were used as controls for *bArKO* mice and wild type (WT) mice served as controls for *tArKO* mice. 2-tailed Student’s *t* test, **P* < 0.05, n=5 per group. Body weight was assessed weekly from 3 to 48 weeks of age in (**C**) female *bArKO* and (**D**) female *tArKO* mice. 2-tailed Student’s *t* test, **P* < 0.05, n=7-8 for controls, n=8 for *bArKO* mice, n=7 for WT and *tArKO* mice.

**Figure S2 Memory for novel object recognition is not different among old *bArKO* and *tArKO* mice of both sexes.** Novel object recognition (NOR) testing was performed in old *bArKO* mice (**A** and **C**) and old *tArKO* mice (**B** and **D**) of both sexes at the age of >19 months. Discrimination index for time exploring the novel object was calculated for old *bArKO* (**A**) and *tArKO* (**B**) mice. Discrimination index for interactions with the novel object was calculated for old *bArKO* (**C**) and *tArKO* (**D**) mice. The number of mice used in the test is indicated in the graph. Two-way ANOVA with Tukey’s multiple comparison test was used.

**Figure S3 Memory for social recognition is not different among old *bArKO* and *tArKO* mice of both sexes.** Social recognition (SR) testing was performed in old *bArKO* mice (**A** and **C**) and old *tArKO* mice (**B** and **D**) of both sexes at the age of >19 months. The experimental mouse was videotaped for 10 min; SR was measured by (**A** and **B**) the number of interactions with as well as (**C** and **D**) the time spent around the “old” and “new” intruder mice. For *bArKO* mice, n=9 for control males, n=5 for *bArKO* males, n=6 control females, and n=5 *bArKO* females. For *tArKO* mice, n=8 for males and WT females and n=6 for *tArKO* female mice. Two-way ANOVA with Tukey’s multiple comparison test was used.

**Figure S4 Whole body aromatase knockout and decreased estrogen production are associated with low locomotor activity (LA) in old female *tArKO* mice.** LA was measured in (**A**) young and (**B**) old *bArKO* mice as well as (**C**) young and (**D**) old *tArKO* mice. The number of mice used in the test is indicated in each column. Two-way ANOVA with Tukey’s multiple comparison test was used, **P* < 0.05, ***P* < 0.01, *****P* < 0.0001.

**Figure S5 Whole-body total aromatase knockout may link to anxiety-like behavior in old female *tArKO* mice.** The open field test (OF) was performed in young and old *bArKO* mice (**A** and **B**) and young and old *tArKO* mice (**C** and **D**) of both sexes. Less time spent in and fewer entries into the center of the apparatus indicate anxiety-like behavior. Old female *tArKO* mice showed fewer entries into the center of the apparatus than WT mice. The number of mice used in the test is indicated in each column. Two-way ANOVA with Tukey’s multiple comparison test was used. **P* < 0.05.

**Figure S6 *bArKO* and *tArKO* mice do not exhibit anxiety-like behavior in the light/dark box test**. The light-dark box test (LD) was performed in old *bArKO* and old *tArKO* mice of both sexes at age > 19 months. We measured time in the light compartment of the box (**A, C**) and entries into the light compartment (**B, D**) for *bArKO* mice (**A, B**) and *tArKO* mice (**C, D**). There was no difference between all groups for time spent in or the number of entries into the light compartment of the box. Mice spent less time and had fewer entries into the light compartment of the box suggesting higher levels of anxiety-like behavior. The number of mice used in the test is indicated above each column in the graph. Two-way ANOVA with Tukey’s multiple comparison test was used.

**Figure S7** **Bulk RNA-seq analysis of hippocampi shows no significant dysregulation between young *bArKO* and littermate control (Ctrl) mice of both sexes.** Volcano plot showing differential gene expression between (**A**) young male *bArKO* and (**B**) young female *bArKO* and their sex-matched littermate control mice using Wald test.

**Figure S8** **Bulk RNA-seq analysis of hippocampi from *bArKO* male mice across age.** (**A**) Volcano plot displaying differential expression (DE) between old male *bArKO* and littermate control (Ctrl) mice using Wald test. (**B**) Boxplots showing differentially expressed genes (DEGs) between old male *bArKO* and Ctrl mice; counts normalized using the median of ratios method. P values are calculated using Wald test and adjusted using Benjamini-Hochberg correction. (**C**) Pathway enrichment analysis (over-representation analysis) of DEGs by age for male *bArKO* mice only using GO MF and cellular component (CC) sets. Up to the top 10 most significantly enriched sets per ontology are shown. P values are calculated using hypergeometric test. (**D**) Expression heatmap of DEGs by age in male *bArKO* mice only. Values are counts normalized using the median of ratios method, scaled across rows.

**Figure S9 Validation of ECM-related proteins and SLC17A6 in *bArKO* and *tArKO* mice by immunofluorescent staining.** (**A**-**C**) Immunofluorescence staining was performed to assess the expression of ECM-related proteins (CCN2 [**A**], DCN [**B**]) and SLC17A6 (**C**) in the brains of aged *bArKO* and *tArKO* mice (n = 3-4 per group). Co-immunostaining with GFAP and NeuN was conducted to identify astrocytes and neurons, respectively. IgG immunostaining served as a negative control. (**D**-**F**) Quantification of immunofluorescence signal intensity of CCN2 [**D**], DCN [**E**], and SLC17A6 (**F**) in the hippocampal CA1 region in control (Ctrl)/WT, *bArKO,* and *tArKO* mice. Kruskal-Wallis with Dunn's multiple comparisons test was used. Scale bars, 100 µM.

**Figure S10 Major ECM regulating pathways, TGFβ and Wnt, are not significantly altered in the hippocampus of old female *bArKO* and *tArKO* mice.** (**A**) Normalized RNA counts of TGFβ and Wnt ligands between aged female control (Ctrl) and *bArKO* hippocampus by bulk RNA-seq. (**B**) Normalized RNA counts of TGFβ receptors 1 and 2 (*Tgfbr1*, *Tgfbr2*) between aged female Ctrl and *bArKO* hippocampus by bulk RNA-seq. Immunoblotting showed expression (left) and quantification (right) of TGFBR2 and WNT1 in the hippocampus of *bArKO* (**C**) *and tArKO* (**D**) female mice at old age. β-actin served as the loading control. n=3-4. WT, wild type. The Mann-Whitney test was used.

**Figure S11 ER expression in the hippocampus of *bArKO* mice.** mRNA levels of *ERα (***A***)*, *ERβ (***B***)*, and *Gper1* (**C**) in the hippocampus of young (6 months) and old (>19 months) *bArKO* mice of both sexes. Ratios of ERα/ERβ (**D**) and ERα/Gper1 (**E**) are calculated. *Gapdh* mRNA level served as the loading control. n=3. Two-way ANOVA with Tukey’s multiple comparison test. (**F**) Immunoblotting showed ERα expression (top) and quantification (bottom) in the hippocampus of old *bArKO* mice of both sexes (~ 20 months). β-actin served as the loading control. n=3-4. ♂, males and ♀, females.

**Figure S12. Hippocampal cell-type specific expression of estrogen receptors and differentially expressed ECM genes identified in the mouse hippocampus in the Allen Brain Cell (ABC) transcriptomic atlas.** (**A**) UMAP of 176k hippocampus cells from adult mouse brain from the ABC atlas, colored by broad cell type. (**B**-**C**) UMAPs of cells from (A) colored for estrogen receptors (**B**) and differentially expressed ECM genes (**C**).

**Figure S13 Differentially expressed ECM genes from the hippocampal astrocytes are not significantly altered between wild-type and astrocyte ERβ conditional knockout (*ERβ cKO*) mice (GSE220288).** (**A**) Schematic of experimental design. At 12 months of age, the hippocampus was collected for bulk RNA-seq from wild-type (WT) and *ERβ cKO* mice. (**B**) Normalized RNA counts of differentially expressed ECM genes (*Ccn2*, *Col1a1*, *Dcn*, *Ogn*) between WT and ERβ cKO female hippocampus. All comparisons are not significant (*P* > 0.05). Wald test through DESeq2 was used.
